# piRNA-46403 Promotes Sertoli Cell Ferroptosis via Disrupting m5C-dependent USP18 mRNA Stabilization in Varicocele-mediated Infertility

**DOI:** 10.7150/ijbs.137298

**Published:** 2026-08-12

**Authors:** Xiao Fang, Li Song, Dong Liu, Zhixiang Xin, Wenjuan Pang, Qiang Tong, Jing Huang, Anbang Wang, Lei Yin, Hejing Huang, Aimin Jiang, Shancheng Ren

**Affiliations:** 1 Department of Urology, 905th Hospital of PLA Navy, Shanghai, China.; 2 Department of Urology, Changzheng Hospital, Naval Medical University (Second Military Medical University), Shanghai, China.; 3 Reproductive Medicine Center, Changzheng Hospital, Naval Medical University (Second Military Medical University), Shanghai, China.; 4 Department of Ultrasound, Naval Medical Center, Shanghai, China.; 5 Department of Ultrasound, Changzheng Hospital, Naval Medical University (Second Military Medical University), Shanghai, China.; 6 Department of Urology, Changhai Hospital, Naval Medical University (Second Military Medical University), Shanghai, China.

**Keywords:** piRNA-46403, m5C modification, Sertoli cells, ferroptosis, spermatogenesis, varicocele

## Abstract

Varicocele (VC) represents a primary etiology of male infertility, yet the epigenetic landscapes and molecular triggers driving VC-associated spermatogenic failure remain largely enigmatic. Herein, it is demonstrated that Sertoli cell ferroptosis is a critical pathological feature of VC, and its pharmacological inhibition effectively rescues VC-induced reproductive impairment *in vivo*. Through small RNA sequencing of clinical testicular tissues, piRNA-46403 is identified as a significantly upregulated mediator in VC patients with asthenospermia. Functionally, piRNA-46403 is shown to orchestrate Sertoli cell ferroptosis, whereas its silencing attenuates spermatogonial apoptosis and restores spermatogenic function. Mechanistically, piRNA-46403 acts as a molecular decoy that binds to the RNA-binding protein YBX1. This interaction disrupts the binding of YBX1 to m5C-modified USP18 mRNA, thereby reducing USP18 mRNA stability and triggering ferroptosis. These findings elucidate a novel piRNA-46403/YBX1/m5C-modified USP18 regulatory axis in the male reproductive system and position piRNA-46403 as a promising therapeutic target for mitigating VC-mediated infertility.

## Introduction

Varicocele (VC) is a vascular disease characterized by the abnormal dilation, elongation, and convolution of the spermatic veins, leading to pain, discomfort, and progressive testicular dysfunction 1. VC is one of the most common causes of male infertility, affecting approximately 15-20% of the general male population. The prevalence of VC increases to about 35% in men with primary infertility and rises to 70-80% in those with secondary infertility 2, 3. In recent years, surgical treatment, particularly microsurgical varicocelectomy, has become the primary treatment modality. Microsurgical varicocelectomy can improve the rate of sperm recovery in azoospermic patients with VC and enhance pregnancy and live birth rates in oligospermic patients with VC 4. Nevertheless, while varicocelectomy is effective in ameliorating sperm quality and clinical outcomes 5, the molecular mechanisms underlying VC-associated infertility remain unclear. Therefore, elucidating the molecular mechanisms by which VC results in abnormal spermatogenesis is of great significance for the personalized diagnosis and treatment of male infertility.

Ferroptosis is a novel form of iron-dependent regulated cell death triggered by the accumulation of lethal lipid peroxides 6. Emerging evidence implicates ferroptosis as a contributing factor in male reproductive disorders. For instance, ferroptosis has been shown to drive asthenospermia and impair sperm function 7. Suppression of ferroptosis mitigated oligospermia in male Nrf2 knockout mice 8. Salidroside ameliorated oligoasthenospermia by suppressing ferroptosis through inhibition of the NF-κB pathway 9. Moreover, excessive ferroptosis of Sertoli cells contributes to spermatogenic impairment. Transferrin receptor is a ferroptosis-related marker that can regulate the ferroptosis of Sertoli cells, making it a hopeful therapeutic target for treating male infertility 10. However, the molecular mechanisms of Sertoli cell ferroptosis in male infertility remain largely elusive, posing a significant challenge for developing targeted therapies for VC.

Various small non-coding RNAs have been reported to be associated with male infertility, such as microRNAs (miRNAs), transfer RNA-derived small RNAs (tsRNAs), and PIWI-interacting RNA (piRNA) 11, 12. miRNA-31-5p mediated the proliferation and apoptosis of human spermatogonial stem cells by targeting JAZF1 and Cyclin A2 13. Upregulation of miRNA-10a in germ cells induced male infertility through targeting Rad51 in both mice and humans 14. Circulating exosomal tRF-Glu-CTC-005 and tRF-Gly-GCC-002 could serve as predictive factors for the success of microdissection testicular sperm extraction in patients with non-obstructive azoospermia 15. In recent years, the critical roles of piRNAs in mammalian spermatogenesis have garnered increasing attention. piRNAs form complexes with PIWI proteins to ensure the normal progression of spermatogenesis, from spermatogonial proliferation and meiosis to sperm maturation, by silencing transposons, regulating chromatin remodeling, and modulating the translation of sperm-specific mRNAs 16, 17. Mutations in genes involved in the piRNA pathway, such as PIWIL, MOV10L1, and TDRD, can lead to reduced piRNA levels and transposon derepression, resulting in spermatogenic arrest or non-obstructive azoospermia 18-20. Additionally, piRNAs are modulated by environmental factors and epigenetic regulation during spermatogenesis; consequently, their alterations may serve as potential biomarkers for the diagnosis of male infertility and the development of future intervention strategies 12. However, the specific regulatory roles and molecular mechanisms of piRNAs in VC-associated spermatogenic dysfunction remain to be elucidated.

Sertoli cells play a crucial role in spermatogenesis, providing nutrients and structural support to germ cells, maintaining the blood-testis barrier and the homeostasis of the testicular microenvironment 21. Abnormalities in Sertoli cell function can amplify the damaging effects of VC-related microcirculation disorders, oxidative stress, and metabolic disturbances on the spermatogenic process 22, 23. Currently, the molecular mechanisms by which Sertoli cell abnormalities mediate infertility under VC conditions remain unclear, especially at the level of piRNAs and RNA modifications related to reproduction. Therefore, we have chosen Sertoli cells as the research entry point, aiming to discover new mechanisms and molecular targets. In this study, we aimed to investigate the role and underlying mechanisms by which piRNAs regulate ferroptosis in Sertoli cells and their contribution to VC-mediated infertility. Small RNA sequencing was performed to profile piRNA expression in testicular tissues from normal controls (NC), VC patients with normal semen (VN), and VC patients with asthenospermia (VA). Functional assays in cells and animal models were conducted to elucidate the roles and mechanisms of piRNAs in Sertoli cell ferroptosis. Our study provides new insights into the treatment of male infertility.

## Materials and Methods

### Clinical sample collection

Testicular tissues, serum and semen were obtained from five NC subjects, seven VN patients, and ten VA patients in Shanghai Changzheng Hospital. The NC group consisted of five testicular tumor patients undergoing radical orchiectomy. Prior to surgery, all subjects underwent semen analysis and exhibited semen parameters within the WHO normal reference ranges. Semen cryopreservation was performed for future fertility preservation. Semen and serum samples were collected preoperatively, and histologically normal adjacent (non-tumor) testicular tissues were obtained during surgery. Testicular biopsies were taken from VN patients and VA patients. All participants offered their informed consent. **Table S1** lists the results of semen analyses of the NC, VN, and VA groups. The samples were stored at -80°C for the subsequent study.

### Ethics statement

Human studies were approved by the Medical Ethics Committee of Changzheng Hospital, Naval Medical University (Second Military Medical University). Written informed consent was obtained from all participants. Animal experiments were approved by the Laboratory Animal Ethics Committee of Changzheng Hospital, Naval Medical University (Second Military Medical University).

### Measurement of serum hormone levels

Serum concentrations of estradiol (E2), luteinizing hormone (LH), and follicle-stimulating hormone (FSH) were quantified using enzyme-linked immunosorbent assay (ELISA). Commercial ELISA kits were used, including the human E2 ELISA kit (CB10447-Hu), human LH ELISA kit (CB10454-Hu), and human FSH ELISA kit (CB10458-Hu). All procedures were carried out in accordance with the manufacturers' protocols. Optical density was measured at 450 nm, and hormone levels were calculated from standard calibration curves.

### Establishment of VC rat model and treatment

Thirty male SD rats (210 ± 20 g, aged 12 weeks) were obtained from SPF (Beijing) Biotechnology Co., Ltd. (China). The rats were randomly divided into 3 groups: normal control (NC) group, VC group, and VC+ferroptosis inhibitor (Fer-1) group, with 10 rats in each group. For the induction of VC, rats were anaesthetised by intraperitoneal injection of ketamine (75 mg/kg) and xylazine (2.5 mg/kg), and VC was induced by utilizing the Turner's method 24. An incision was made along the midline of the abdomen using surgical scissors. The abdominal contents were then moved to the right side of the body using moistened gauze. A self-made metal probe rod with a diameter of about 0.8mm was placed parallel to the running direction of the renal vein above the left renal vein, and then the left renal vein and the metal probe rod were ligated together with 4-0 silk thread. The left renal vein filled rapidly and expanded in diameter due to impaired venous outflow after ligation. Then the metal probe was carefully removed, by which time the ligation site of the left renal vein has been narrowed to about half of its original size before the surgery. The NC group experienced the same procedure, but the left renal vein was not ligated. After 1 week of recovery, the rats in the VC+Fer-1 group were intraperitoneally injected with Fer-1 (0.7mg/kg) twice a week, and the other groups were injected with equal volume normal saline. After 4 weeks, the rats were anesthetized, and the testicles and epididymis were removed. Testicular mass index was calculated according to testicular wet weight/body weight × 100%. The work has been reported in accordance with the ARRIVE guidelines (Animals in Research: Reporting *In Vivo* Experiments) 25.

To investigate the role of piRNA-46403 in VC, rats were divided into the NC group, the VC group, and the VC+piRNA-46403 agomir group (5 rats each group). The NC and VC groups were the same as described above. piRNA-46403 agomir treatment commenced one week after the induction of VC and continued for 4 weeks. Rats were administered piRNA-46403 agomir (1 nmol/g/day) 26 via tail vein injection twice weekly, while other groups received an equivalent volume of saline. Four weeks later, the rats were anesthetized and euthanized, and their testes were harvested.

### Hematoxylin-eosin (HE) staining

The testicular tissues were fixed, embedded, and sectioned into slices of 5 μm in thickness. Subsequently, these sections were dewaxed using xylene. A graduated series of alcohols was employed to hydrate the sections. After undergoing a hematoxylin and eosin staining process, the slices were dehydrated and sealed. The prepped slides were carefully analyzed using a microscope.

### Serum biochemical analysis

After fasting for 12 h, blood samples were collected from rats and centrifuged to obtain serum. Serum levels of alanine aminotransferase (ALT), aspartate aminotransferase (AST), alkaline phosphatase (ALP), total bilirubin (TB), creatinine (Cr), and blood urea nitrogen (BUN) were measured using commercial assay kits purchased from COIBO BIO (China): rat ALT (CB10236-Ra), AST/GOT (CB10802-Ra), ALP (CB11480-Ra), TB (CB11077-Ra), Cr (CB10454-Ra), and BUN (CB10659-Ra), according to the manufacturers' instructions. Absorbance was measured at 450 nm, and concentrations were calculated based on standard curves.

### GSH, MDA, and Fe2+ detection

The levels of GSH, MDA, and Fe2+ were detected by GSH Assay Kit, MDA Assay Kit (Beyotime, S0131), and Iron Colorimetric Assay Kit (APPLYGEN, #E1042), respectively. The experimental procedures strictly adhered to the guidelines provided by the kit manufacturers. Absorbance was then obtained using a microplate reader (Infinite M1000, TECAN).

### TdT-mediated dUTP Nick-End labeling (TUNEL) staining

The TUNEL assay kit (Beyotime) was utilized to assess cell apoptosis in testicular tissues. In brief, the paraffin-embedded tissue sections were deparaffinized and rehydrated, followed by treatment with 20 µg/mL proteinase K for 20 minutes at room temperature to enhance tissue permeabilization. The tissue sections were then subjected to a one-hour incubation at 37 °C with a biotinylated TUNEL reaction mixture. Subsequently, diaminobenzidine tetrahydrochloride chromogen (Invitrogen) and hematoxylin were used for staining. The stained sections were examined under a microscope (Olympus, Japan).

### Immunofluorescence

Fixed testicular tissues were embedded and cut into 4-μm-thick sections. Afterward, the tissues were fixed on the slides at 60 °C for 30 min. Following PBS washing, sections were subjected to antigen retrieval and permeabilized with 0.3 % Triton X-100 at room temperature, followed by blocking with 3% BSA at room temperature for 30 min. Afterwards, the sections were incubated with anti-GATA4, anti-GPX4, anti-ACSL4, anti-NRF2, and anti-USP18 antibodies overnight at 4 °C. After washing with PBS, the sections were incubated with secondary antibodies for 1 h at room temperature. Finally, Nuclei were counterstained with DAPI and images were captured using a fluorescence microscope.

Immunofluorescence in Sertoli cells was performed as follows. Cells were fixed with 4% paraformaldehyde at room temperature for 30 min. Permeabilization was performed with 0.1% Triton X-100 for 10 min, followed by PBS washes. Cells were blocked with 3% BSA for 30 min and incubated with anti-USP18, and anti-YBX1 anti-SLC7A11 antibodies at 4 °C overnight. After PBS washes, cells were incubated with secondary antibodies at room temperature for 1 h in the dark. Nuclei were counterstained with DAPI, and fluorescent signals were observed using a fluorescence microscope.

### Small RNA sequencing

Total RNA was extracted from testicular tissues of NC, VN, and VA groups using TRIzol reagent (Invitrogen, USA). The RNA quality and concentration were evaluated with a NanoDrop ONE spectrophotometer (Thermo Fisher Scientific, USA), and its integrity was verified by 2% agarose gel electrophoresis. Construction of the small RNA libraries was performed using the Multiplex Small RNA Library Prep Kit (USA). In short, ligand primers were added to the RNA fragments at both ends, and cDNA synthesis was performed through PCR amplification. The PCR products were then analyzed on an 8% SDS-PAGE gel. Sequencing was carried out on the Illumina HiSeq X Ten platform (USA). For the piRNA analysis, sequences that did not map to the miRBase were selected within the length range of 24-33 bp, and then mapped to the piRNAcluster (derived from the piRNAcluster database), to obtain potential piRNAs. Differentially expressed piRNAs were determined using the R package DEseq2, with a threshold set at |log2 (foldchange)| > 1 and P-value< 0.05. Analysis of gene ontology (GO) and Kyoto Encyclopedia of Genes and Genomes (KEGG) pathway enrichment for the target mRNAs of piRNAs was carried out with a p-value threshold of 0.05.

### Quantitative real-time PCR (qRT-PCR)

Total RNA was extracted using TRIzol Reagent (Invitrogen, USA) following the manufacturer's instructions. RNA concentrations were measured with a NanoDrop 2000 spectrophotometer (Thermo, USA). For cDNA synthesis, 1 µg of RNA from each sample was reverse-transcribed using the RevertAid First Strand cDNA Synthesis Kit (Thermo, K1622) according to the manufacturer's protocol. qRT-PCR was performed on an ABI Q6 Real-Time PCR System (Applied Biosystems, USA) in a 20 µL reaction volume using SYBR Green Master Mix (Roche). Relative gene expression levels were calculated using the 2^-ΔΔCt method. Primer sequences used in this study are listed in **Table S2.**

### Cell culture

Sertoli cells were obtained from iCell Bioscience (China), and spermatogonial cells were procured from Procell (China). The cells were cultured in specialized complete medium containing 10% fetal bovine serum (GIBCO) and 1% penicillin/streptomycin with 5% CO2 at 37 °C. To simulate VC microenvironmental stress *in vitro*, Sertoli cells were subjected to hypoxic treatment (1% O2, 48h).

### Cell transfection

piRNA-46403 inhibitor, piRNA-46403 mimic, USP18 siRNA, YBX1 overexpressing plasmid, DNMT1 overexpressing plasmid, and DNMT1 siRNA were designed and synthesized by GenePharma (Shanghai, China). Transfections were then conducted using Lipofectamine 3000 (Invitrogen) following the manufacturer's instructions.

### Fluorescence in situ hybridization (FISH) combined with immunofluorescence

Paraffin-embedded testicular tissues were sectioned at 5 μm, baked at 60 °C, deparaffinized, and rehydrated. Endogenous peroxidase activity was quenched with 3% H_2_O_2_. After pepsin digestion to expose target RNA fragments, sections were pre-hybridized and then incubated with digoxigenin-labeled piRNA-46403 probes at 40 °C overnight. Following SSC washes, sections were incubated with biotinylated anti-digoxigenin antibody and FITC-conjugated streptavidin-biotin complex. Subsequently, immunofluorescence staining was performed by blocking sections and incubating with primary antibodies anti-GATA4, anti-CD31, anti-GCNA1 at 4 °C overnight, followed by fluorophore-conjugated secondary antibodies. Nuclei were counterstained with DAPI, and sections were examined under a fluorescence microscope.

FISH combined with immunofluorescence in Sertoli cells was performed as follows. Cells were fixed with 4% paraformaldehyde at room temperature, washed with DEPC-treated water, adjusted to a density of 1×10^6^ cells/mL, followed by baking at 56 °C. Endogenous peroxidase activity was quenched with 3% H_2_O_2_, and cells were digested with pepsin to expose target RNA fragments. After pre-hybridization, cells were incubated with digoxigenin-labeled piRNA-46403 or USP18 probes at 40 °C overnight. Following stringent SSC washes, cells were sequentially incubated with blocking solution, biotinylated anti-digoxigenin antibody, and FITC-conjugated streptavidin-biotin complex. Subsequently, immunofluorescence staining was performed by blocking with BSA, incubating with primary antibodies anti-YBX1 at 4 °C overnight, and then with secondary antibodies. Nuclei were counterstained with DAPI, and slides were observed under a fluorescence microscope.

### Cell Counting Kit-8 (CCK-8)

To assess cellular viability, the CCK-8 was utilized. Briefly, cells were plated into 96-well plates at a density of 6×10^3^ cells per well. After hypoxic treatment for 0, 24, 48, and 72 h, 20 μL of CCK-8 solution was added to each well and incubated for 2 hours. The absorbance value at 450 nm was then measured using a microplate reader (Thermo, USA).

### Transmission electron microscope (TEM)

TEM was used to examine Sertoli cellular ultrastructure. Sertoli cells were collected and resuspended in TEM fixative solution at 4 °C. Afterward, the cells were pre-embedded in agarose, followed by a 2-hour fixation process at ambient temperature with 2.5% glutaraldehyde. After sequential dehydration in graded alcohols, the samples were then impregnated and solidified with a mixture of propylene oxide and epoxy resin. The sections were subsequently cut, stained, and examined under a TEM.

### ROS detection

The ROS levels in Sertoli cells were measured using the ROS Assay Kit. Briefly, Sertoli cells were seeded into 12-well plates and adhered overnight. Subsequently, 10μM DCFH-DA and Hoechst 33342 were introduced to the wells, followed by a 20-minute incubation period at 37 °C in the dark. After two washes with DMEM, fluorescence signals were captured using a fluorescence microscope. The fluorescence intensity of DCFH-DA was analyzed using Image J software.

### Western blot

Protein was isolated from the Sertoli cells using RIPA lysis buffer. Protein separation was achieved via sodium dodecyl sulfate-polyacrylamide gel electrophoresis (SDS-PAGE) and transferred onto polyvinylidene fluoride (PVDF) membranes (Millipore, USA). After a 2-hour blockade with 5% non-fat milk, the membranes were probed with the primary antibodies against GAPDH (1:15000, 60004-1-Ig, Proteintech), SLC7A11 (1:1000, 28731-1-AP, Proteintech), GPX4 (1:1000, Sc-166570, Santa), ACSL4 (1:5000, 22401-1-AP, Proteintech), NRF2 (1:5000, 16396-1-AP, Proteintech), USP18 (1:1000, 12153-1-AP, Proteintech), YBX1 (1:15000, 20339-1-AP, Proteintech), and DNMT1 (1:15000, 24206-1-AP, Proteintech) overnight at 4 °C. Following this, the membranes were incubated with the secondary antibodies Goat Anti-Mouse IgG H&L(HRP) (1:5000, RGAM001, Proteintech) or Goat Anti-Rabbit IgG H&L(HRP) (1:5000, RGAR001, Proteintech) for 1.5 hours.

### Flow cytometry

The apoptosis of spermatogonia was assessed using the Annexin V-FITC Apoptosis Detection Kit (Beyotime, China). Cells were grown in 6-well plates and stained with Annexin V-FITC and PI in as per the manufacturer's guidelines for apoptosis analysis. Flow cytometric analysis was conducted on a flow cytometer (BD FACSVerse™), and the resulting data were analyzed using FlowJo software.

### RNA sequencing

Total RNA was extracted from Sertoli cells with piRNA-46403 mimics and control using TRIzol. The quality of RNA was assessed by measuring the A260/A280 ratio using a NanoDrop™ OneC spectrophotometer (Thermo). The integrity of the RNA was verified through electrophoresis on a 1.5% agarose gel. RNA-sequencing was conducted on the Illumina HiSeq 2500 platform. Differentially expressed genes were detected utilizing the DESeq2 R package (version 3.0.3), applying a significance cutoff of p-value ≤ 0.05 and an absolute log2 fold change threshold of ≥ 1. Analysis of GO and KEGG pathway enrichment for the differentially expressed genes was carried out using the clusterProfiler R package (version 3.0.3), with a p-value threshold of 0.05, to produce the enrichment bubble plots.

### RNA pull down and mass spectrometry

The lysates, along with biotinylated piRNA-46403, were mixed with magnetic beads (Invitrogen, USA) and incubated overnight at 4 °C. Subsequently, the beads were subjected to four washes with lysis buffer. The RNA-protein complexes were then denatured in SDS buffer and subjected to mass spectrometry analysis using the Q Exactive (Thermo Fisher) and Easy-nLC 1000(Thermo Fisher). The raw file from mass spectrometry was searched using the Proteome Discoverer 2.5 engine to retrieve the corresponding database, and finally, the identified protein results were obtained.

### Molecular docking analysis

The three-dimensional structure of YBX1 was downloaded from the Protein Data Bank (PDB) database (https://www.rcsb.org/). The x3dna website (http://web.x3dna.org/fibermodel/rnaseq) was utilized to build the RNA 3D structure model. Subsequently, the protein-RNA molecular docking was performed utilizing the HDOCK server (http://hdock.phys.hust.edu.cn/).

### RNA pull-down

The RNA pull-down assay was conducted using the Magnetic RNA-Protein Pull-Down Kit (Thermo Fisher Scientific) in accordance with the manufacturer's instructions. Briefly, biotin-labeled USP18 RNA probe was incubated with magnetic beads, and then the RNA-bound beads were incubated with Sertoli cell lysates. Finally, the beads were washed and boiled in sodium dodecyl sulfate buffer to retrieve proteins for western blot analysis.

### RNA immunoprecipitation (RIP)

Magnetic beads were mixed with 5 µg of anti-YBX1 (20339-1-AP, Proteintech) or 1µg of anti-IgG, the treated magnetic beads were added to Sertoli cell lysates, and the mixtures were incubated at 4 °C for 12 h. Next, proteinase K buffer was added to digest the complexes for 30 min at 55 °C. After that, buffer was employed to extract the RNA. The extracted RNA was subsequently analyzed by RT-qPCR. IgG was used as a negative control.

### Methylated RNA immunoprecipitation (MeRIP)

Total RNA was extracted from Sertoli cells by TRIzol reagent and then immunoprecipitated with magnetic beads precoated with 10 μg of anti-m5C antibody (Abcam, ab214727), or anti-IgG (Abcam, ab172730). After immunoprecipitation, proteinase K digestion buffer (Invitrogen, USA) was added and incubated at 55 °C for 30 min. After washes, RNA was isolated, and RT-qPCR was performed as described previously.

### Dual-luciferase reporter assay

The m5C modification sites of USP18 were predicted using RMBase v3.0. The predicted m5C site of USP18 was mutated, and corresponding wild-type and mutant luciferase reporter vectors were constructed, named USP18-WT or USP18-MUT, respectively. Subsequently, the vectors were co-transfected into Sertoli cells with YBX1 overexpression. Luciferase activity was measured 48 hours post-transfection.

### mRNA stability assay

To evaluate USP18 mRNA stability in Sertoli cells, we treated the cells with 5 μg/mL of actinomycin D. Then, the cells were harvested at 0, 2, 4, 8, 12h, and total RNA was isolated for qRT-PCR to determine mRNA stability.

### Co-immunoprecipitation (Co-IP)

To investigate the interaction between USP18 and SLC7A11, Sertoli cells were lysed using IP lysis buffer. Lysates were pre-cleared with protein A/G beads at 4 °C for 4 h. Subsequently, immunoprecipitation was performed overnight at 4 °C using protein A/G beads coupled with antibodies against USP18, SLC7A11, or IgG. The immunoprecipitated complexes were then washed with IP lysis buffer and analyzed by western blot.

### Protein stability assay

To assess SLC7A11 protein stability, Sertoli cells were treated with cycloheximide (CHX). Cells were harvested at the indicated time points (0, 2, 4, 6, and 8 h), and total protein was extracted. The remaining SLC7A11 protein levels were analyzed by western blot.

### Statistical analysis

Statistical analysis was conducted utilizing GraphPad Prism version 9.0 software. Comparisons between two groups were conducted with a two-tailed Student's t-test, whereas one-way analysis of variance (ANOVA) was applied for comparisons involving more than two groups. A p-value below 0.05 was deemed statistically significant.

## Results

### Ferroptosis occurs in Sertoli cells of VC patients with asthenospermia

In clinical practice, VC is closely associated with testicular dysfunction. Three representative cases of testicular elastography were evaluated using the Mindray® Resona 7 system with the shear wave elastography technique. **Figures S1A** and S**1B** illustrated an NC subject with WHO-normal semen parameters, showing approximately equivalent elasticity in both testes. **Figures S1C and S1D** depicted a left-sided VC patient (resting diameter: 2.2mm) with normal sperm parameters, showing reduced testicular stiffness on the VC-affected side. **Figures S1E and S1F** present a case of left-sided VC (resting diameter: 2.7 mm) accompanied by oligoasthenoteratozoospermia (OAT), where the affected testicle demonstrated slightly higher stiffness compared to the contralateral, unaffected side. These findings suggest that the severity of VC may be associated with alterations in testicular morphology, tissue elasticity and physiological function.

**Figure 1:**
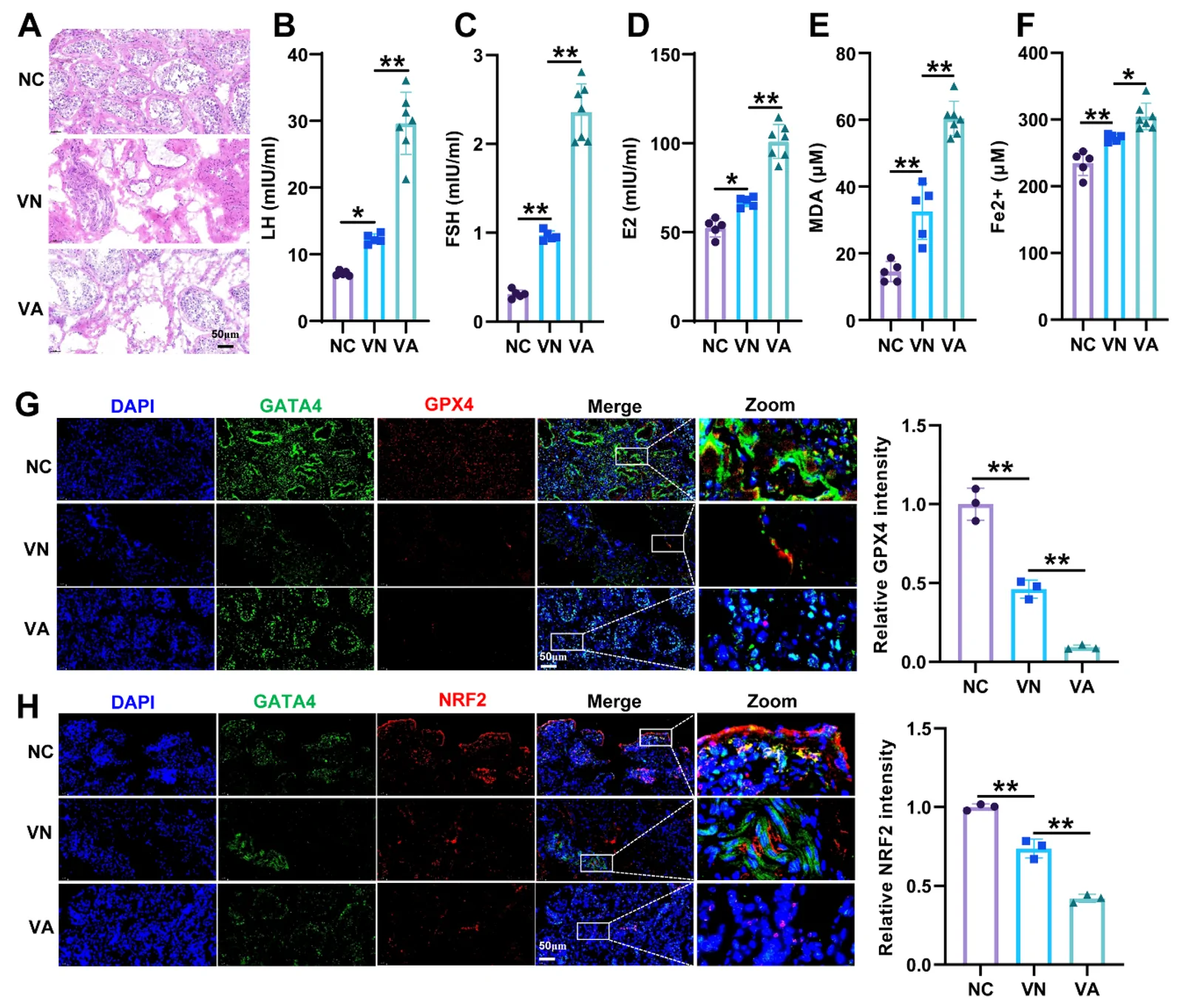
** Ferroptosis occurs in Sertoli cells of patients with varicocele (**VC)**.** (A) HE staining image of testicular tissues in VC patients. (B-D) Levels of luteinizing hormone (LH), follicle-stimulating hormone (FSH), and estradiol (E2) were quantified in serum samples from VC patients by enzyme-linked immunosorbent assay (ELISA). (E) Lipid peroxidation in serum was evaluated by determining malondialdehyde (MDA) content using a commercial assay kit. (F) Fe2+ level in serum was assessed by with an iron ion detection kit. The co-localization of Sertoli cell marker GATA4 and ferroptosis marker GPX4 (G) and ferroptosis-related antioxidant regulator NRF2 (H) in testicular tissues was detected by immunofluorescence double staining. Testicular tissues and serum samples were collected from three groups: NC (testicular tumor patients who exhibited semen parameters within the WHO normal reference ranges preoperatively, with normal adjacent non-tumor testicular tissues obtained during surgery and serum samples collected preoperatively), VN (VC patients with normal semen), and VA (VC patients with asthenospermia). Scale bar = 50μm. *P<0.05; **P < 0.01.

To further understand the pathological characteristics of patients with VC, we collected testicular tissues from NC, VN, and VA patients. HE staining revealed that spermatogenic cells in the NC group had distinct layers, clear morphological structure, and a large number of sperm in the lumen (**Figure 1A**). In the VN group, spermatogenic cells had clear morphology, but the structure of seminiferous tubules was damaged. In the VA group, spermatogenic cells exhibited irregular morphology, loose arrangement, and reduced sperm count. Serum analyses showed that levels of LH, FSH, and E2 were significantly elevated in the VN group and further increased in the VA group, reflecting an exacerbation of spermatogenic dysfunction in VA patients (**Figure 1B-D**). Similarly, serum MDA and Fe2+ levels were increased in the VN group and exhibited a further rise in the VA group, indicating the presence of ferroptosis in VA patients (**Figure 1E, 1F**). Immunofluorescence double staining showed that ferroptosis marker GPX4 and ferroptosis-related antioxidant regulator NRF2 were separately co-localized with the Sertoli cell marker GATA4 in testicular tissues. Meanwhile, the expression of GPX4 and NRF2 was remarkably decreased in the VN group and further decreased in the VA group (**Figure 1G, 1H**). Overall, ferroptosis occurs in Sertoli cells of VC patients with asthenospermia.

### Inhibition of ferroptosis alleviates VC-mediated spermatogenic dysfunction in rats

**Figure 2:**
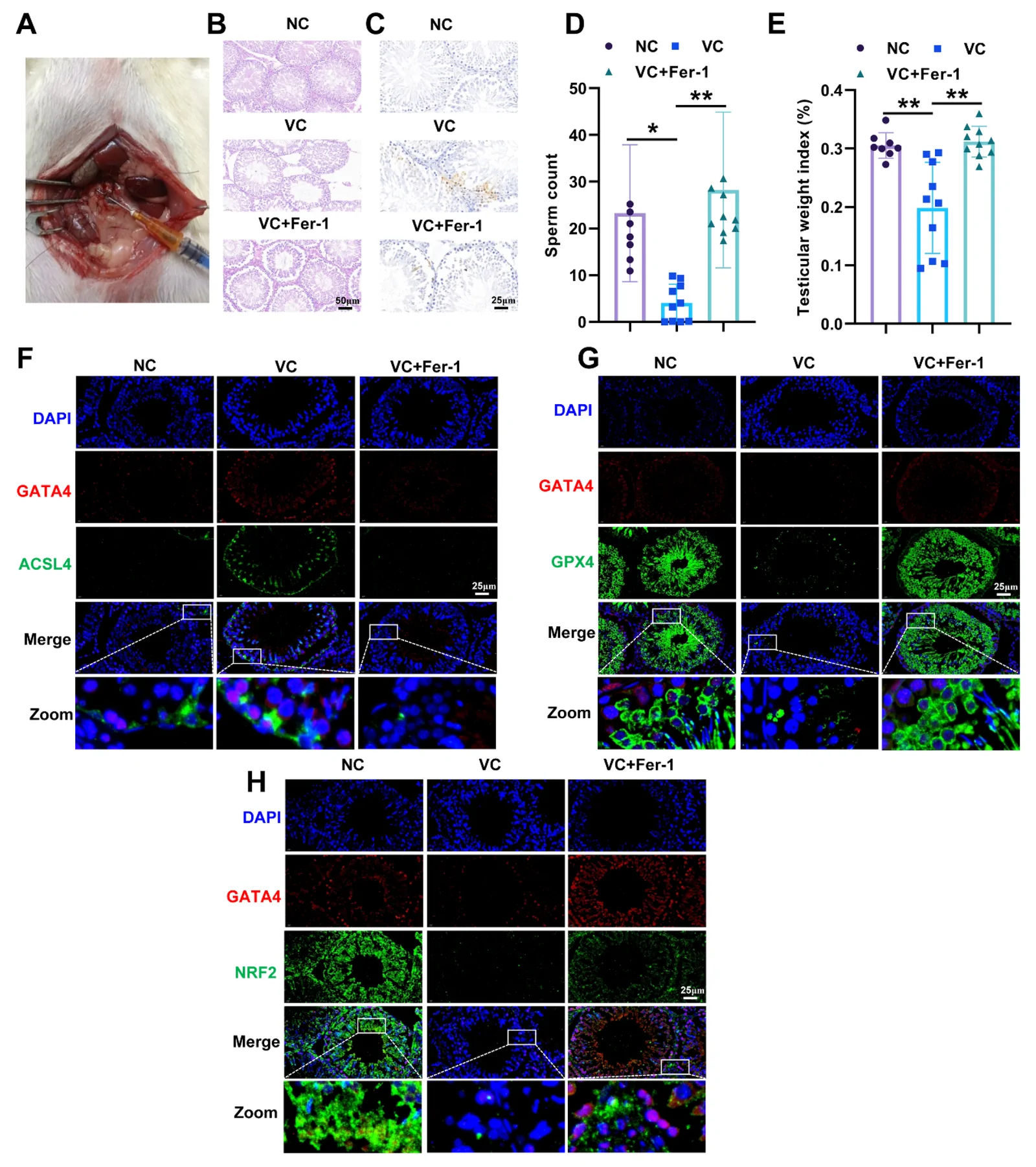
** Inhibition of ferroptosis alleviates rat spermatogenic dysfunction caused by varicocele (VC).** (A) The construction of VC rat model. (B) HE staining of testicular tissues in NC, VC, and VC+Fer-1 groups. Scale bar = 50μm. (C) Apoptotic cells in testicular tissues from NC, VC, and VC+Fer-1 groups were visualized by TUNEL staining. Scale bar = 25μm. (D) Sperm count was determined to evaluate spermatogenic capacity. (E) Testicular weight index was calculated to assess testicular function. The co-localization of Sertoli cell marker GATA4 and ferroptosis marker ACSL4 (F), GPX4 (G) and ferroptosis-related antioxidant regulator NRF2 (H) in testicular tissues was detected by immunofluorescence double staining. Scale bar = 25μm. *P < 0.05; **P < 0.01.

To investigate the role of ferroptosis in VC-mediated infertility *in vivo*, we established a VC rat model (**Figure 2A**). HE staining of testicular tissues revealed that spermatogenic cells in the control group had distinct layers, clear morphological structure, and there was a lot of sperm in the lumen (**Figure 2B**). In the VC group, spermatogenic cells displayed irregular morphology, loose arrangement, and reduced sperm count. Treatment with the ferroptosis inhibitor Fer-1 largely preserved spermatogenic cell integrity and restored sperm numbers. TUNEL staining further revealed an increased number of apoptotic cells in the VC group compared with controls, whereas Fer-1 treatment decreased apoptosis (**Figure 2C**). Consistently, sperm count and testicular weight index were significantly reduced in the VC group but were substantially restored following Fer-1 administration (**Figure 2D, 2E**). Immunofluorescence double staining showed that ferroptosis markers ACSL4, GPX4 and ferroptosis-related antioxidant regulator NRF2 were expressed in GATA4-positive Sertoli cells in testicular tissues (**Figure 2F-H**). In parallel, the expression of ACSL4 was increased, while the expression of GPX4 and NRF2 was decreased in the VC group. However, Fer-1 treatment showed opposite trends (**Figure 2F-H**). Taken together, ferroptosis inhibition mitigates VC-induced spermatogenic dysfunction in rats.

### piRNA-46403 is upregulated in testicular tissues of VC patients with asthenospermia

**Figure 3:**
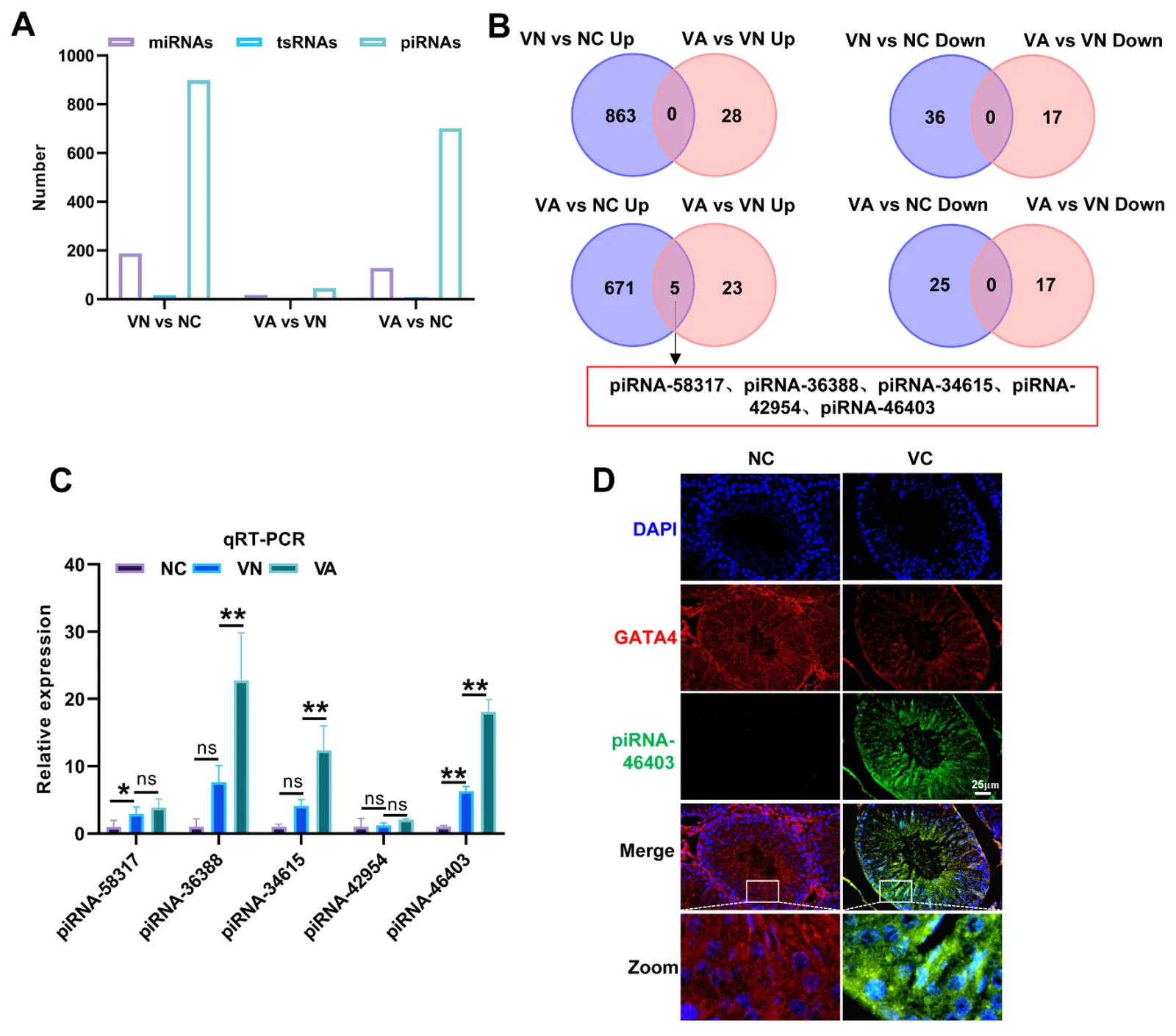
** piRNA-46403 is upregulated in testicular tissues of varicocele (**VC) **patients.** (A) The number of differentially expressed small RNAs was determined by small RNA sequencing in testicular tissues from NC, VN, and VA patients. (B) Venn diagram analysis of commonly upregulated or downregulated differentially expressed piRNAs between different groups. (C) qRT-PCR validation of candidate piRNA expression in testicular tissues from NC, VN, and VA patients. (D) Immunofluorescence and FISH were used to detect co-localization of piRNA-46403 and GATA4 in testicular tissues of NC, and VC groups. Scale bar = 25μm. ns, not significant, *P < 0.05; **P < 0.01.

To screen for key piRNAs involved in VC-mediated infertility, we conducted small RNA sequencing in testicular tissues from NC, VN, and VA patients. The results revealed that among all small RNAs, piRNAs exhibited the most significant differences in expression (**Figure 3A, Figure S2A-S2C**). There were 899 differentially expressed piRNAs between the NC and VN groups, 701 differentially expressed piRNAs between the NC and VA groups, and 45 differentially expressed piRNAs between the VN and VA groups (**Figure S2C, S3A-S3C**). The GO analysis of target genes of differentially expressed piRNAs indicated that the target genes in the VA vs NC group and the VA vs VN group were primarily associated with cell adhesion, transport, and cell differentiation (**Figure S3D, S3E**). KEGG analysis revealed that the target genes in the VA vs NC group and the VA vs VN group were mainly involved in the Wnt, Rap1, and MAPK signaling pathways (**Figure S3F, S3G**). The Venn diagram analysis showed that only the VA vs NC group and the VA vs VN group had 5 commonly upregulated piRNAs (**Figure 3B**). Therefore, qRT-PCR validation in human testicular tissues was performed on these 5 piRNAs. The result showed that piRNA-46403 was the most significantly upregulated in the VA group (**Figure 3C**). Therefore, piRNA-46403 was chosen as the subject for subsequent research. Sequence analysis revealed that piRNA-46403 is a 31-nt human piRNA located on chromosome 15 (chr15:101762373-101762404) **(Figure S4A)**. Immunofluorescence combined with FISH using piRNA-46403 probe, Sertoli cell marker GATA4, spermatogonia marker GCNA1, and endothelial cell marker CD31 showed that piRNA-46403 expression was upregulated in Sertoli cells of the VC group compared with the NC group, but remained unchanged in spermatogonia and endothelial cells, implicating piRNA-46403 as a key regulator in Sertoli cells (**Figure 3D, S4B, S4C**). In conclusion, piRNA-46403 was upregulated in testicular tissues of VC patients with asthenospermia.

### piRNA-46403 inhibits the cell viability of spermatogonia by promoting Sertoli cell ferroptosis

**Figure 4:**
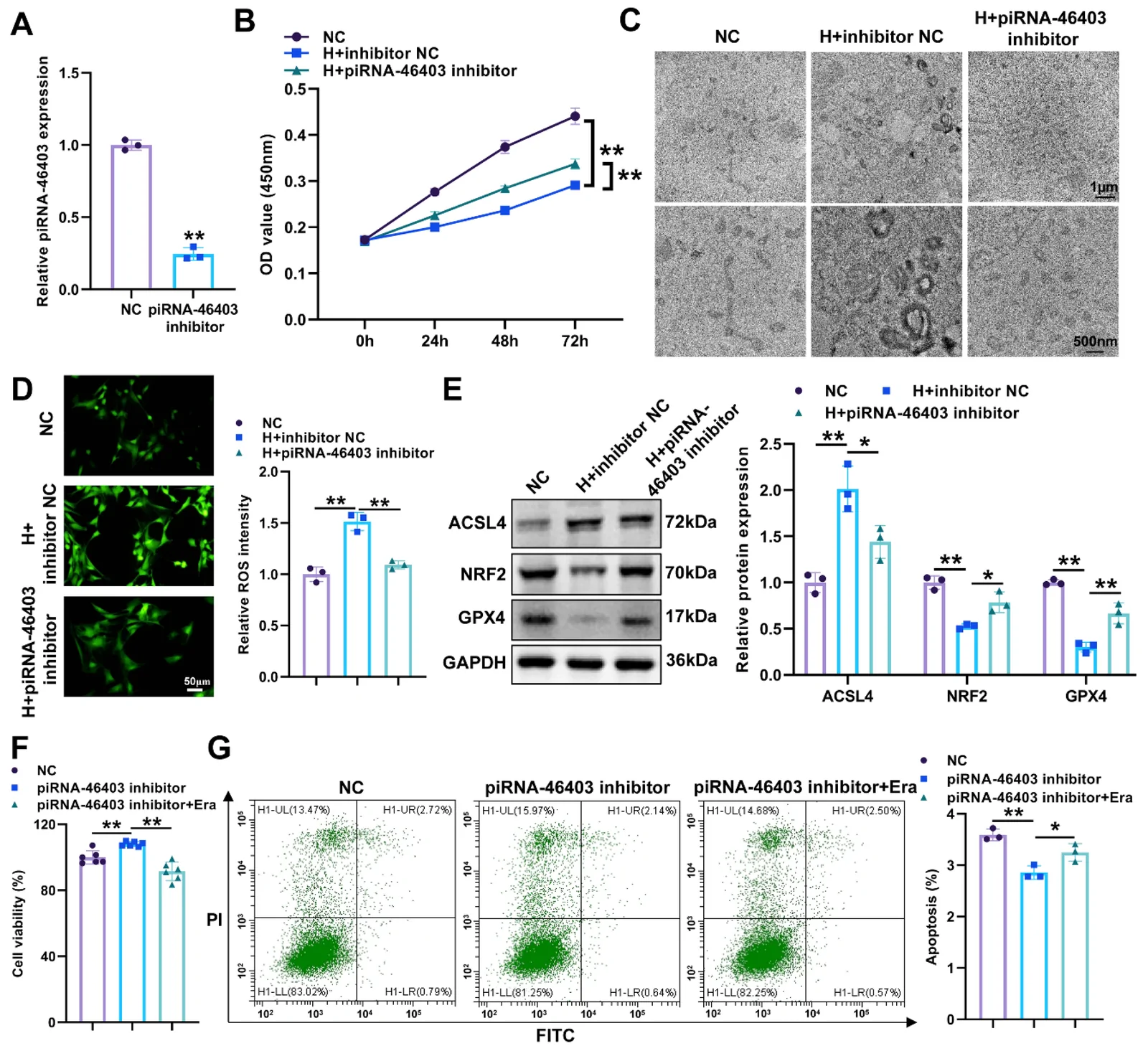
** piRNA-46403 promotes Sertoli cell ferroptosis.** (A) qRT-PCR analysis of piRNA-46403 knockdown efficiency in Sertoli cells. (B) The CCK-8 assay was employed to evaluate Sertoli cell proliferation following piRNA-46403 knockdown. (C) Evaluation of ferroptosis-associated morphology in piRNA-46403-knockdown Sertoli cells via transmission electron microscopy. (D) The level of ROS in Sertoli cells with piRNA-46403 knockdown was evaluated using the DCFH-DA probe. Scale bar=50μm. (E) Western blot analysis of ACSL4, NRF2, and GPX4 expression in Sertoli cells after piRNA-46403 knockdown. (F, G) CCK-8 (F) and flow cytometry (G) were utilized to assess cell viability and apoptosis of spermatogonia incubated with supernatants from piRNA-46403 knockdown Sertoli cells treated with erastin, respectively. NC represents normal control. H represents hypoxia. Era represents erastin. *P < 0.05; **P < 0.01.

To investigate the regulatory role of piRNA-46403 in Sertoli cell ferroptosis *in vitro*, Sertoli cells were subjected to hypoxic treatment and transfected with a piRNA-46403 inhibitor. The knockdown efficiency of piRNA-46403 was confirmed by qRT-PCR (**Figure 4A**). CCK-8 assays demonstrated that hypoxia significantly reduced the cell viability of Sertoli cells, which was partially rescued by piRNA-46403 inhibition (**Figure 4B**). TEM revealed that hypoxia induced mitochondrial damage with disordered cristae, whereas treatment with the piRNA-46403 inhibitor partially restored mitochondrial structure (**Figure 4C**). ROS assays revealed that hypoxia obviously increased the fluorescence intensity of ROS in Sertoli cells, while piRNA-46403 inhibitor suppressed this effect (**Figure 4D**). Western blot analysis showed that the expression of ACSL4 was significantly elevated, whereas NRF2 and GPX4 were significantly reduced in the hypoxia group. After treatment with piRNA-46403 inhibitor, this effect was reversed (**Figure 4E**). Collectively, these results suggest that piRNA-46403 augmented Sertoli cell ferroptosis. Subsequently, to further investigate the impact of piRNA-46403-induced Sertoli cell ferroptosis on spermatogonia, we incubated spermatogonia with supernatants from Sertoli cells transfected with piRNA-46403 inhibitor and treated with erastin, a ferroptosis activator. CCK-8 and flow cytometry assays revealed that spermatogonia incubated with supernatants from piRNA-46403-knockdown Sertoli cells exhibited significantly enhanced proliferation and reduced apoptosis; however, these effects were counteracted by erastin (**Figure 4F, 4G**). Overall, piRNA-46403-mediated Sertoli cell ferroptosis contributes to reduced spermatogonial cell viability.

### piRNA-46403 induces spermatogenic dysfunction and enhanced Sertoli cell ferroptosis in VC rats

**Figure 5:**
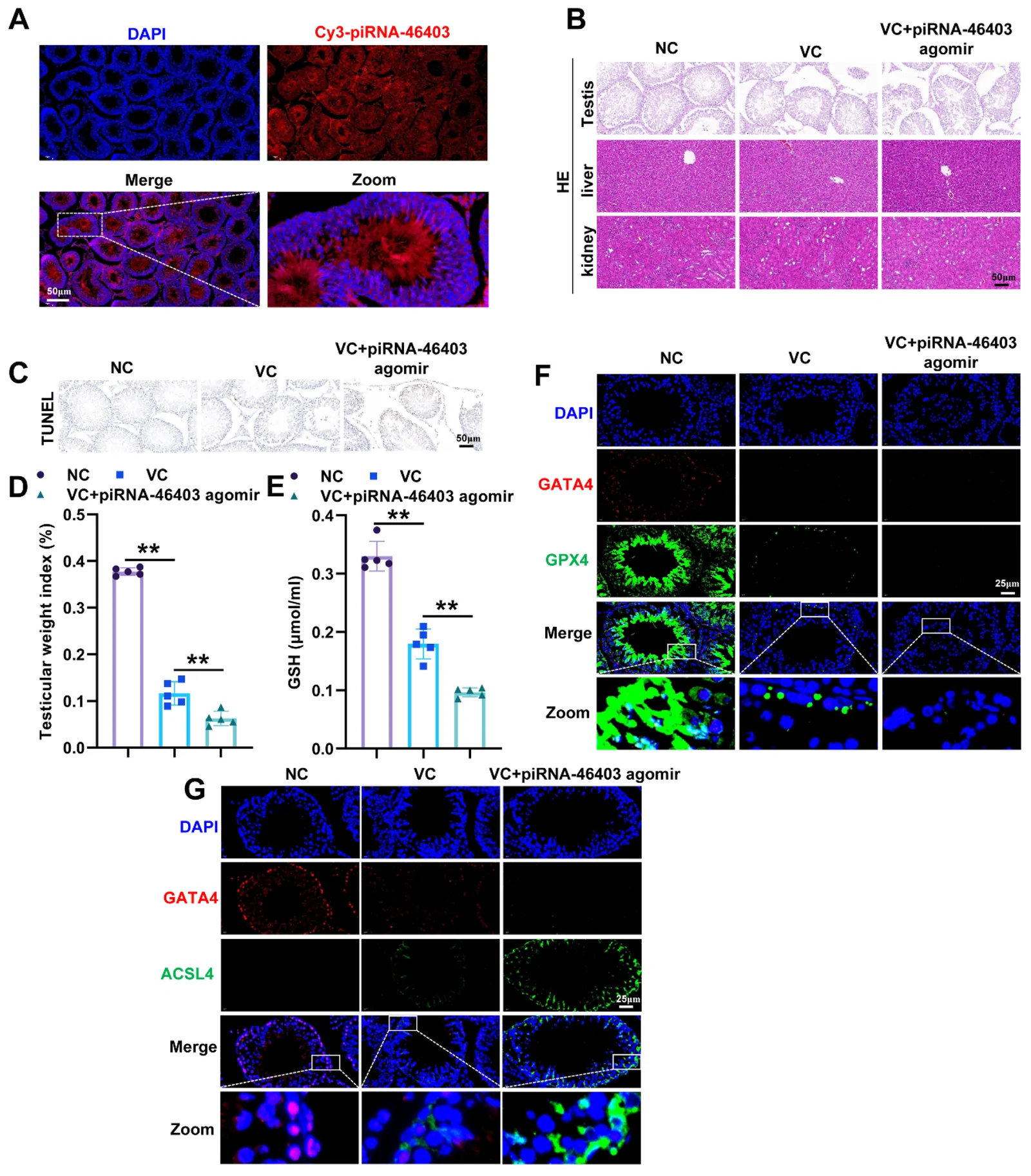
** piRNA-46403 aggravates rat spermatogenic dysfunction caused by varicocele (**VC)**.** (A) Fluorescence tracing was used to observe the expression of piRNA-46403 in rat testicular tissues. Scale bar = 50μm. (B) HE staining images of testicular, liver and kidney tissues from NC, VC, and VC+piRNA-46403 agomir groups. Scale bar = 50μm. (C) TUNEL staining images of testicular tissues in NC, VC, and VC+piRNA-46403 agomir groups. Scale bar = 50μm. (D) Testicular weight index. (E) The level of GSH was detected by the commercial assay kit. The co-localization of Sertoli cell marker GATA4 and ferroptosis markers GPX4 (F), and ACSL4 (G) in testicular tissues was detected by immunofluorescence double staining. Scale bar = 25μm. **P < 0.01.

To investigate the effect of piRNA-46403-mediated Sertoli cell ferroptosis on the impairment of spermatogenesis in rats with VC, we treated VC model rats using piRNA-46403 agomir. Fluorescence microscopy demonstrated accumulation of the Cy3-labeled piRNA-46403 agomir within testicular tissues **(Figure 5A)**, verifying effective delivery and enrichment of piRNA-46403 agomir in testicular tissues. HE staining showed that compared to the control group, the VC group had a decreased sperm count, and piRNA-46403 agomir treatment further reduced the sperm count (**Figure 5B**). However, histological analysis of liver and kidney tissues showed normal architecture among all groups (**Figure 5B**). TUNEL staining indicated that the VC group exhibited a higher count of apoptotic cells relative to the control group, and the administration of piRNA-46403 agomir led to a further rise in the number of apoptotic cells (**Figure 5C**). Moreover, testicular weight index was dramatically decreased in the VC group, and further decreased after piRNA-46403 agomir treatment (**Figure 5D**). To rule out potential systemic toxicity, serum levels of liver function markers (ALT, AST, ALP, and TB) and kidney function markers (BUN and Cr) were measured, and no significant differences were observed among the control, VC, and VC+piRNA-46403 agomir groups (**Figure S5A-5F**). These above results indicated that piRNA-46403 led to impaired spermatogenesis *in vivo*. To further determine whether ferroptosis is involved in the function of piRNA-46403 *in vivo*, we examined ferroptosis indicators. The GSH level was significantly diminished in the VC group, and further reduced in the piRNA-46403 agomir group (**Figure 5E**). Immunofluorescence double staining of ferroptosis markers GPX4 and ACSL4 and Sertoli cell marker GATA4 showed that the expression of GPX4 decreased in the VC group, and further decreased after piRNA-46403 agomir treatment (**Figure 5F**). The opposite results were observed in ACSL4 expression (**Figure 5G**). In summary, piRNA-46403 aggravates spermatogenic dysfunction and promotes Sertoli cell ferroptosis in VC rats.

### piRNA-46403 downregulates USP18 expression in Sertoli cells

**Figure 6:**
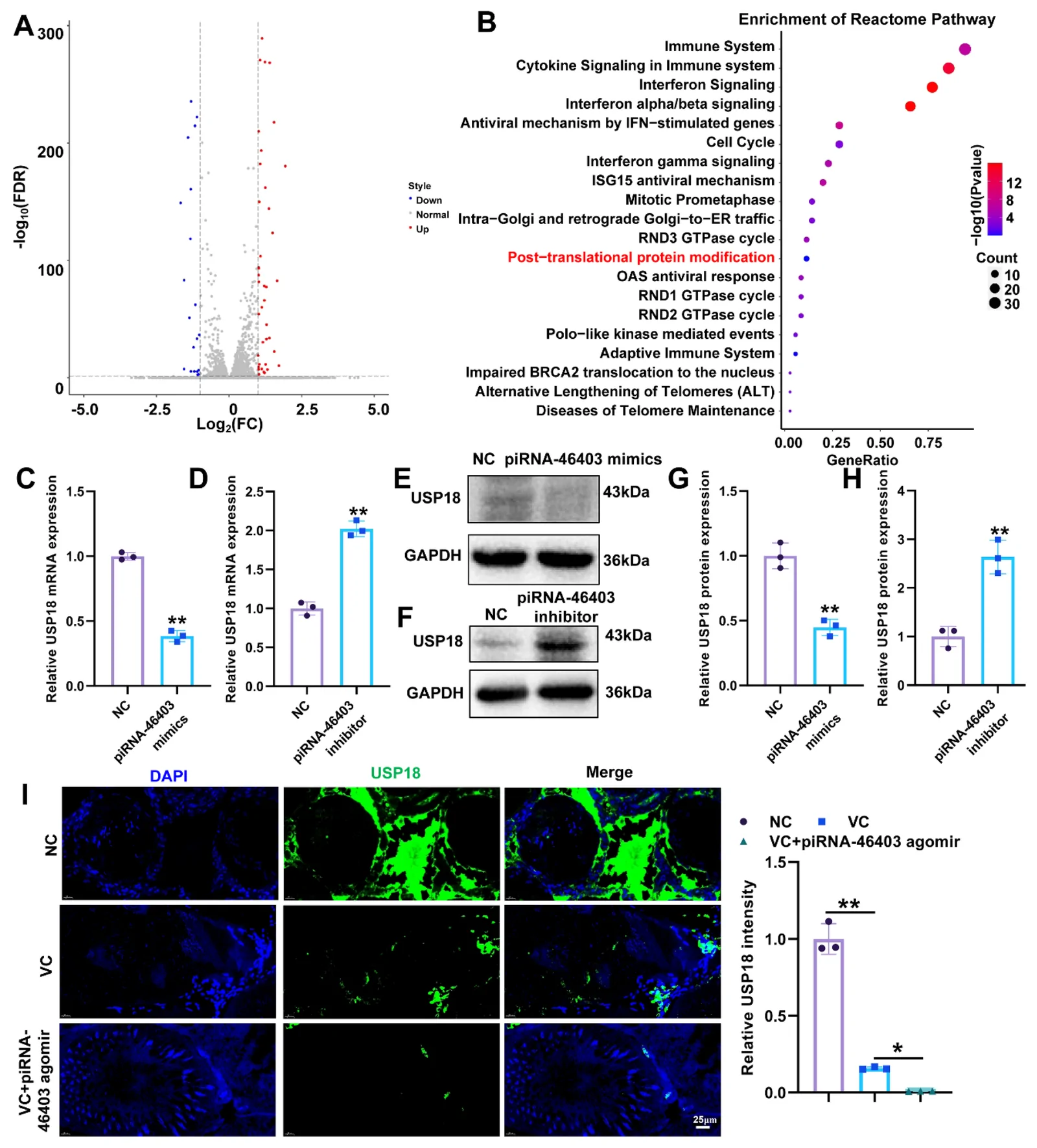
** piRNA-46403 downregulates USP18 expression in Sertoli cells.** (A) Volcano plot showing differentially expressed genes in control and piRNA-46403 mimics Sertoli cells. (B) Reactome pathway analysis of differentially expressed genes regulated by piRNA-46403. (C, D) qRT-PCR analysis was applied to measure the expression of USP18 in Sertoli cells with piRNA-46403 overexpression or knockdown. (E, F) Western blot analysis was applied to measure the expression of USP18 in Sertoli cells with piRNA-46403 overexpression or knockdown. (G, H) Quantity analysis of USP18 protein expression in Sertoli cells. (I) Immunofluorescence detection of the effect of piRNA-46403 agomir on USP18 expression in rat testicular tissues. Scale bar = 25μm. *P < 0.05; **P < 0.01.

piRNAs are a class of novel small non-coding RNAs that play essential roles in male reproduction 27. piRNAs bind to Piwi proteins and play crucial roles in germ cell development by silencing transposable elements and regulating protein-coding genes 17, 28. To investigate whether piRNA-46403 mediates transposon silencing via Piwi proteins, we first analyzed the piRNA-46403 binding proteins identified by RNA pull-down combined with mass spectrometry. We found that Piwi proteins were not present in the resulting list **(Table S3)**. A recent study reported in 2026 that among the multiple transposons detected, the main changes were in different subtypes of LINE1 29, we detected the transposon marker LINE1. qRT-PCR analysis confirmed a significant increase in piRNA-46403 expression in Sertoli cells transfected with piRNA-46403 mimics (**Figure S6A**). No significant difference in LINE1 mRNA expression was observed between the piRNA-46403 mimics and NC groups (**Figure S6B**). Furthermore, methylation-specific qRT-PCR analysis revealed that piRNA-46403 overexpression did not remarkably alter LINE1 methylation levels at the promoter region or gene body regions (GB1, GB2) (**Figure S6C**), indicating that piRNA-46403 may function mainly through non-classical and non-PIWI-dependent pathways. Accumulating evidence indicates that piRNAs also exert post-transcriptional regulatory roles by modulating mRNAs 30. To determine the downstream regulated gene of piRNA-46403, we performed RNA sequencing on Sertoli cells from the control and piRNA-46403 overexpression groups. The volcano plot showed that, compared to the control group, the piRNA-46403 overexpression group had 63 differentially expressed genes, including 41 upregulated and 22 downregulated genes (**Figure 6A**). Subsequently, we conducted a Reactome signaling pathway enrichment analysis on the differentially expressed genes induced by overexpression of piRNA-46403. The results showed that the most significantly enriched pathways included Interferon signaling, Immune system, and Post-translational protein modification (**Figure 6B**). Emerging studies showed post-translational modifications, including ubiquitination, play pivotal roles in regulating protein stability, activity, and interactions, ultimately influencing both the buildup of iron and lipid peroxidation 31, 32. Therefore, we focused on the genes enriched in the Post-translational Protein Modification. We found that USP18, which can regulate protein deubiquitination, was significantly downregulated by piRNA-46403. The latest research indicated that USP18 can induce the deubiquitination of SLC7A11, promoting its protein stability, and ultimately promoting cystine uptake and inhibiting ferroptosis 33. To date, there have been no reports on the role of USP18 in Sertoli cells, infertility, or VC-mediated infertility. Therefore, we chose USP18 for the next research. qRT-PCR and western blot assays indicated that piRNA-46403 overexpression remarkably suppressed the mRNA and protein expression of USP18 in Sertoli cells, respectively, whereas piRNA-46403 silencing exerted the opposite effects (**Figure 6C-H**). Immunofluorescence showed that USP18 expression was reduced in the testicular tissues of VC rats, and piRNA-46403 agomir further promoted the decrease of USP18 expression (**Figure 6I**). These above results revealed that piRNA-46403 inhibited USP18 expression in Sertoli cells.

### piRNA-46403 induces ferroptosis in Sertoli cells by inhibiting USP18

**Figure 7:**
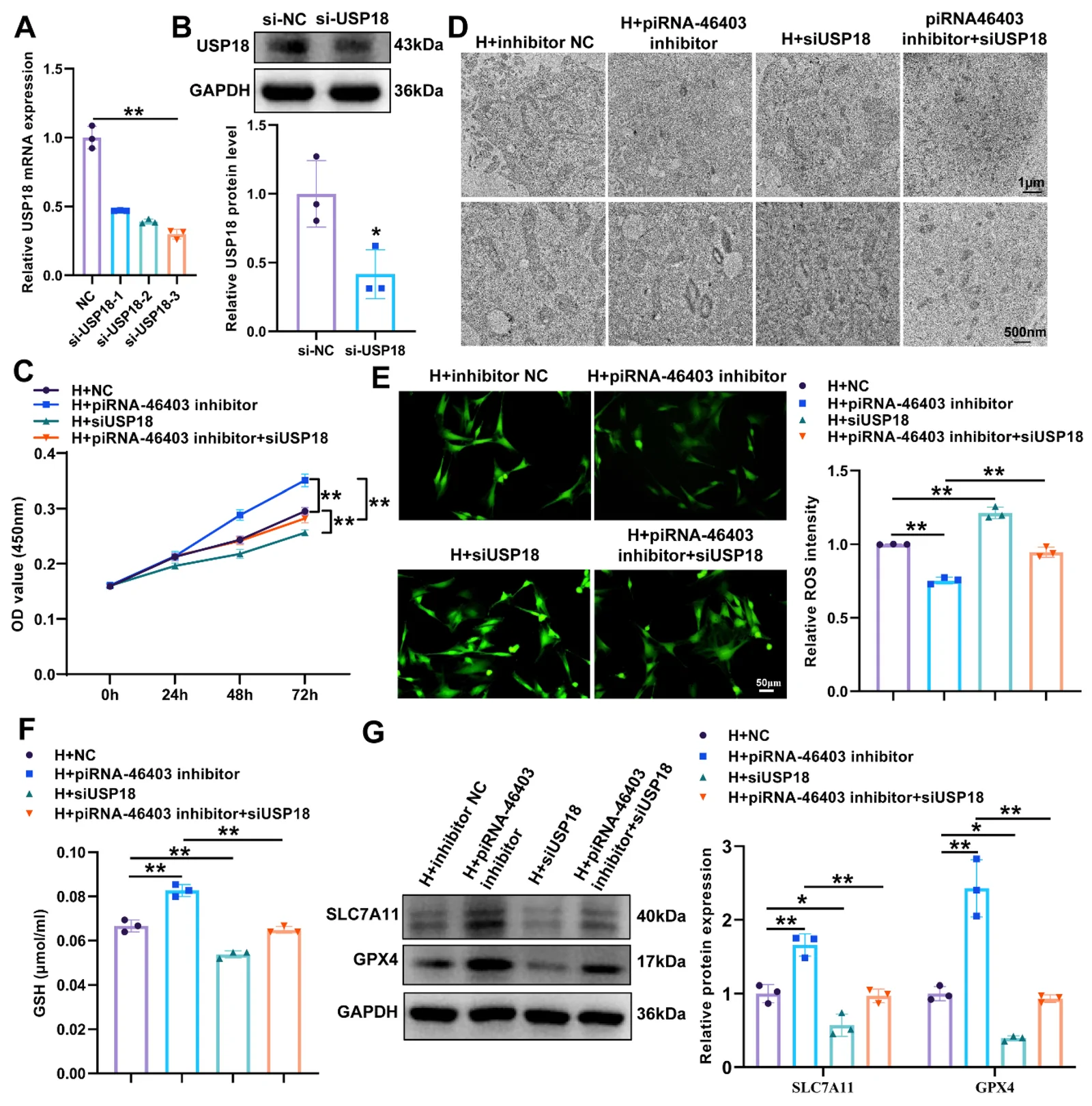
** piRNA-46403 induces ferroptosis in Sertoli cells by inhibiting USP18.** (A, B) qRT-PCR and western blot analysis of USP18 knockdown efficiency in Sertoli cells. (C) The effect of USP18 knockdown on the proliferation of Sertoli cells with piRNA-46403 knockdown was assessed by the CCK-8 assay. (D) Transmission electron microscopy was performed to evaluate the morphological changes in piRNA-46403-knockdown Sertoli cells following USP18 knockdown. (E) ROS levels in piRNA-46403-knockdown Sertoli cells with USP18 knockdown were measured using the DCFH-DA probe. Scale bar = 50μm. (F) The GSH level in piRNA-46403 knockdown Sertoli cells with USP18 knockdown was detected by the commercial GSH assay kit. (G) Western blot analysis of SLC7A11 and GPX4 expression in Sertoli cells with knockdown of both piRNA-46403 and USP18. H represents hypoxia. siUSP18 represents USP18 knockdown. *P < 0.05; **P < 0.01.

To unveil whether piRNA-46403 regulated Sertoli cell ferroptosis through USP18, we knocked down piRNA-46403 and USP18 in Sertoli cells under hypoxic conditions. The knockdown efficiency of USP18 was confirmed by qRT-PCR and western blot (**Figure 7A, 7B**). CCK-8 results showed that knockdown of USP18 abolished the promotive effect of piRNA-46403 knockdown on Sertoli cell viability (**Figure 7C**). TEM revealed that downregulation of USP18 reversed the recovery of cristae structure caused by piRNA-46403 inhibition (**Figure 7D**). ROS assays revealed that knockdown of USP18 reversed the inhibition of ROS levels in Sertoli cells caused by piRNA-46403 knockdown (**Figure 7E**). The GSH level was remarkably increased in Sertoli cells after piRNA-46403 inhibitor treatment, while USP18 knockdown counteracted this effect (**Figure 7F**). Western blot analysis showed that the promotive effect of piRNA-46403 inhibition on the expression of SLC7A11 and GPX4 was repressed by USP18 knockdown (**Figure 7G**). Overall, piRNA-46403 promoted Sertoli cell ferroptosis by inhibiting USP18.

### piRNA-46403 competitively binds to YBX1 to inhibit USP18 mRNA stability in an m5C-dependent manner

**Figure 8:**
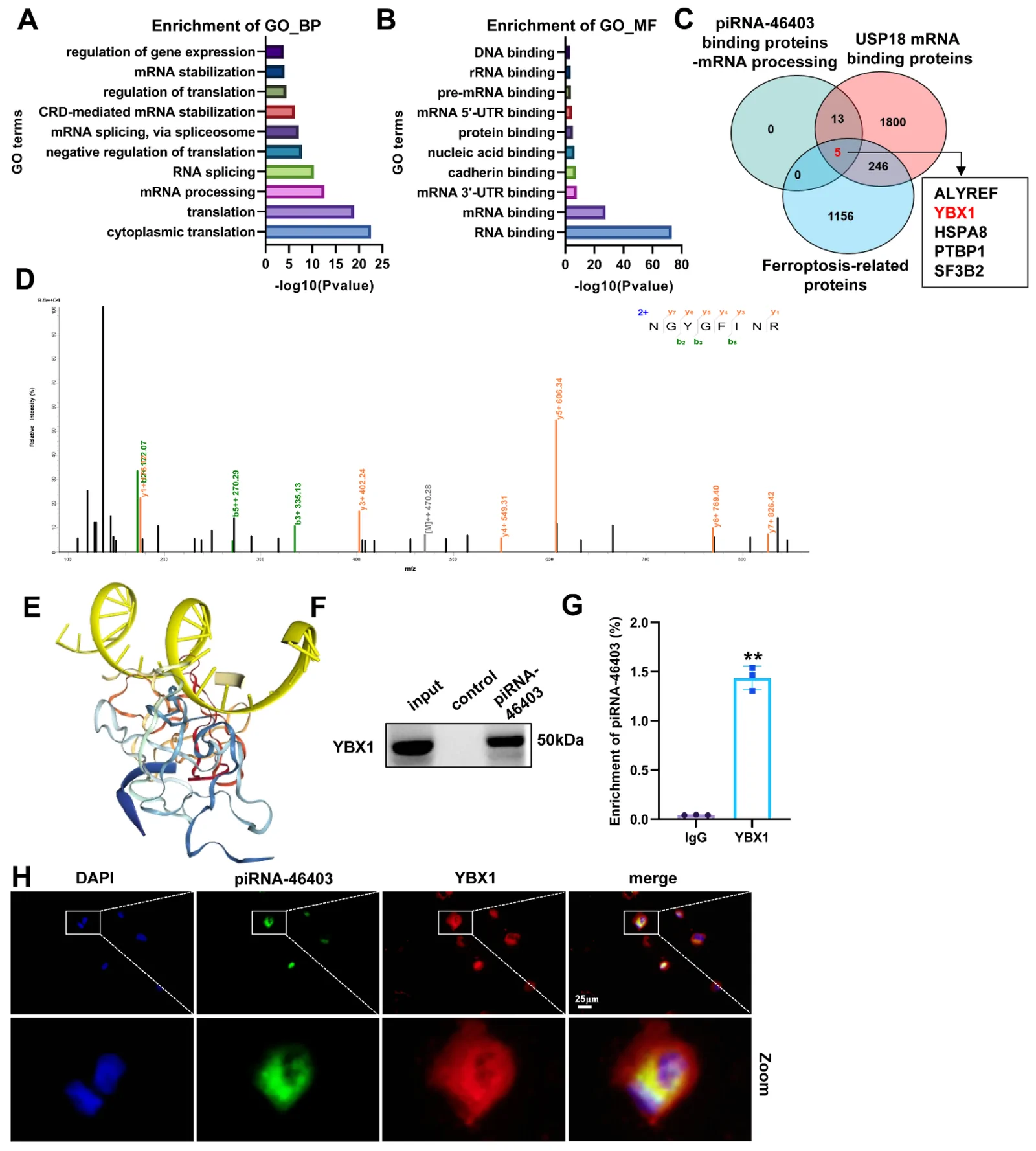
** piRNA-46403 binds to YBX1.** (A, B) GO analysis of the biological processes (A) and molecular functions (B) associated with piRNA-46403-binding proteins. (C) Venn diagram showing the intersection of piRNA-46403 binding proteins involved in mRNA processing, USP18 mRNA binding proteins (from the catRAPID website), and ferroptosis-related genes (from the GeneCards database). (D) Ion flow diagram of YBX1 from mass spectrometry results. (E) Molecular docking analysis of the interaction between piRNA-46403 and YBX1. (F) RNA pull-down combined with western blot verified the binding between piRNA-46403 and YBX1. (G) RIP-PCR analysis of piRNA-46403 enrichment using anti-YBX1 antibodies in Sertoli cells. (H) FISH and immunofluorescence analysis showing the co-localization of piRNA-46403 and YBX1 in Sertoli cells. Scale bar = 25μm. **P < 0.01.

To explore the mechanism by which piRNA-46403 regulated USP18, we used the piRNA-46403 probe for RNA pull-down and mass spectrometry. The results revealed that 184 proteins interacted with piRNA-46403 (**Table S3**). Functional enrichment analysis found that piRNA-46403-targeted proteins were involved in biological processes such as mRNA processing, mRNA stabilization, and RNA splicing (**Figure 8A**); and was enriched in molecular functions like RNA binding and mRNA binding (**Figure 8B**). Since piRNA-46403 primarily regulated the RNA level of USP18, we focused on proteins involved in RNA regulation. We took the intersection of piRNA-46403 binding proteins involved in mRNA processing, USP18 mRNA binding proteins obtained from the catRAPID algorithm analysis, and ferroptosis-related proteins derived from the GeneCards database, yielding 5 proteins (ALYREF, YBX1, HSPA8, PTBP1, and SF3B2) involved in the regulation of ferroptosis (**Figure 8C**). Given that previous studies reported that YBX1 inhibited ferroptosis 34, we focused on YBX1 for further study. The ion flow diagram of YBX1 was shown in Figure 8D. The molecular docking results confirmed the binding of piRNA-46403 to YBX1 (**Figure 8E**). This interaction was further corroborated by RNA pull-down followed by western blot **(Figure 8F)** and RIP-PCR assays** (Figure 8G)**. The FISH and immunofluorescence results showed that piRNA-46403 and YBX1 were co-localized in Sertoli cells (**Figure 8H**). Overall, these findings demonstrate that piRNA-46403 interacts with YBX1.

**Figure 9:**
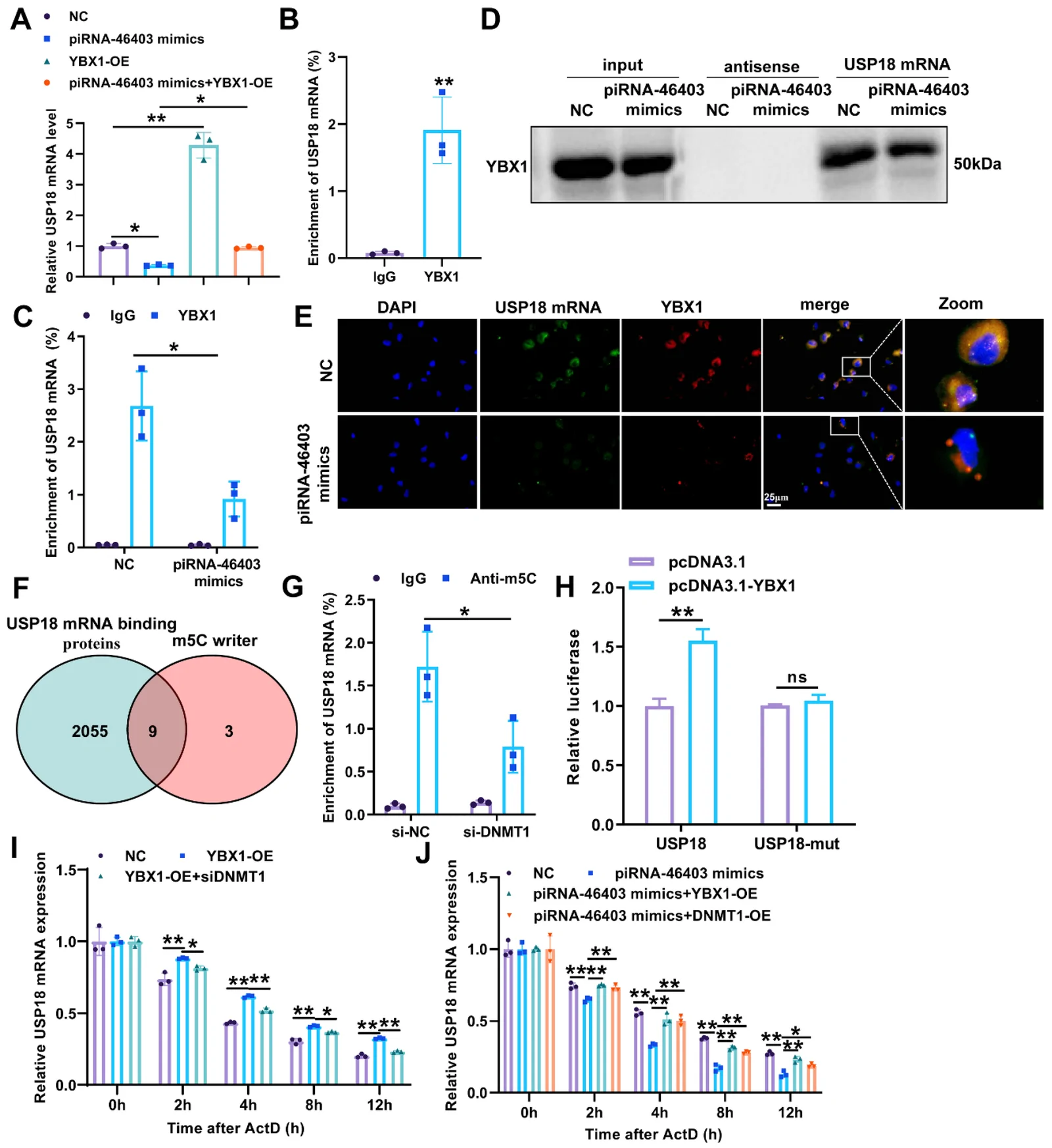
** piRNA-46403 competitively bound to YBX1 to inhibit USP18 in an m5C- dependent manner.** (A) qRT-PCR analysis of USP18 mRNA expression in piRNA-46403 overexpressed Sertoli cells with YBX1 overexpression. (B) RIP-PCR analysis of USP18 mRNA enrichment using anti-YBX1 antibodies in Sertoli cells. (C) RIP-PCR validation of the inhibitory effect of piRNA-46403 on the binding of YBX1 to USP18 mRNA. (D) RNA pull-down using USP18 mRNA probe combined with western blot confirmed that piRNA-46403 inhibits the interaction between YBX1 and USP18 mRNA. (E) FISH and immunofluorescence validation of the inhibitory effect of piRNA-46403 on the binding of YBX1 to USP18 mRNA. Scale bar = 25μm. (F) Venn diagram showing the overlap between USP18 mRNA binding proteins obtained from the catRAPID website and m5C writers. (G) MeRIP-PCR was utilized to assess m5C modification of USP18 after DNMT1 knockdown in Sertoli cells. (H) Dual luciferase reporter assay was used to examine the luciferase activity of wild-type or the mutant USP18 vector in Sertoli cells after YBX1 overexpression. (I) qRT-PCR analysis of USP18 mRNA stability in YBX1 overexpressed Sertoli cells with DNMT1 knockdown. (J) USP18 mRNA stability was measured by qRT-PCR in piRNA-46403-overexpressing Sertoli cells following YBX1 or DNMT1 overexpression. *P < 0.05; **P < 0.01.

Subsequently, we delved into how piRNA-46403 modulates USP18 through YBX1. We first examined the expression of USP18 in Sertoli cells overexpressing both piRNA-46403 and YBX1. We verified the overexpression efficiency of YBX1 via qRT-PCR and western blot **(Figure S7A, S7B)**. The inhibitory effect of piRNA-46403 overexpression on USP18 mRNA expression was reversed by YBX1 overexpression (**Figure 9A**). To further verify whether piRNA-46403 competitively binds to YBX1 to inhibit USP18 mRNA, we performed RIP-PCR assays. The results supported that YBX1 protein bound to USP18 mRNA **(Figure 9B)**, and piRNA-46403 disrupted this YBX1-USP18 mRNA interaction **(Figure 9C).** These findings were also verified by pull down with western blot and immunofluorescence with FISH (**Figure 9D, 9E**). These findings indicate that piRNA-46403 competitively binds to YBX1, thereby inhibiting USP18 mRNA expression.

We then investigated the specific mechanism by which YBX1 regulates USP18 RNA levels. Previous studies have reported that YBX1 relies on m5C modification to enhance the stability of target mRNA, thereby promoting ferroptosis 34. By intersecting m5C writer proteins with the USP18 binding proteins obtained from the catRAPID algorithm, we identified nine potential candidates (**Figure 9F**), among which DNMT1 exhibited the strongest binding ability (**Table S4**). DNMT1 possesses the ability to bind mRNA transcripts and promotes RNA m5C methylation and mRNA stability by recruiting NSUN2 35. We detected m5C modification on USP18 via MeRIP-PCR and found that the m5C modification level decreased following DNMT1 knockdown (**Figure S7C, S7D, 9G**). To determine whether YBX1 modulates USP18 via relying on the m5C modification, we constructed wild-type (WT) USP18 and mutant (MUT) USP18 sequence with the predicted m5C modification sites mutated luciferase reporter plasmids. Luciferase activity assays revealed that YBX1 overexpression obviously increased luciferase activity in the USP18 WT group, but had no significant effect on the USP18 MUT group (**Figure 9H**). These findings indicated that YBX1 bound to USP18 in an m5C-dependent manner. RNA stability assays showed that DNMT1 knockdown counteracted the increased stability of USP18 mRNA caused by YBX1 overexpression (**Figure 9I**). Furthermore, YBX1 and DNMT1 overexpression both neutralized the reduced stability of USP18 mRNA caused by piRNA-46403 overexpression (**Figure S7E, S7F, 9J**). Together, these results indicated that piRNA-46403 competitively bound to YBX1, blocking YBX1 from binding to m5C-modified USP18 mRNA, thereby inhibiting the stability of USP18 mRNA.

Previous studies have reported that USP18 induced the deubiquitination of SLC7A11, thereby enhancing its protein stability and ultimately suppressing ferroptosis 33. Therefore, we hypothesize that piRNA-46403 regulates SLC7A11 protein stability through USP18, thereby promoting ferroptosis in Sertoli cells. To validate this hypothesis, we first examined the interaction between USP18 and SLC7A11. Immunofluorescence analysis revealed colocalization of USP18 and SLC7A11 **(Figure S8A).** We next investigated whether piRNA-46403 regulates the binding between USP18 and SLC7A11. Co-IP showed that piRNA-46403 overexpression reduced the association between USP18 and SLC7A11, which may be attributable to the decreased USP18 expression induced by piRNA-46403 (**Figure S8B**). Protein stability assays demonstrated that piRNA-46403 overexpression significantly reduced the stability of SLC7A11 (**Figure S8C, S8D**). Collectively, these findings indicate that piRNA-46403 suppresses SLC7A11 protein stability via USP18.

## Discussion

VC is a common male clinical condition that mainly refers to the dilation and tortuosity of internal spermatic veins 36, with an overall prevalence of approximately 15% in men 37. Infertility associated with VC arises from the combined effects of multiple factors, including genetic variations, hypoxia, and oxidative stress, rather than a single cause 38. However, the precise mechanisms linking VC to impaired spermatogenesis remain unclear. In this study, piRNA-46403 was upregulated in testicular tissue of VC patients with asthenospermia and inhibited cell viability of spermatogonia by promoting ferroptosis in Sertoli cells. Mechanistically, piRNA-46403 competitively bound to YBX1 to diminish USP18 mRNA stability in an m5C-dependent manner. USP18 knockdown could reverse the inhibitory effect of piRNA-46403 inhibition on Sertoli cell ferroptosis. Collectively, these findings suggest that piRNA-46403 may serve as a potential therapeutic target for VC-mediated infertility (**Figure S9**).

Our elastography findings further support the notion that VC-associated testicular dysfunction is a dynamic and stage-dependent process. In VC patients with normal semen parameters, the affected testis exhibited reduced stiffness compared with the contralateral side, which may reflect early-stage hemodynamic alterations characterized by venous dilatation, blood stasis, and interstitial edema. Such changes can increase vascular compliance and tissue fluid content, thereby reducing apparent shear wave stiffness. In contrast, in VC patients with OAT, the affected testis demonstrated slightly increased stiffness, suggesting a shift toward advanced structural remodeling. Accumulating evidence indicates that VC leads to sustained testicular hypoxia, oxidative stress, and inflammatory activation, which in turn promote testicular tissue injury 39, 40. These progressive histopathological alterations reduce tissue elasticity and increase mechanical stiffness, consistent with our observations in the OAT case 41. Therefore, testicular stiffness assessed by shear wave elastography may reflect the balance between early congestive changes and late fibrotic remodeling, providing a noninvasive surrogate marker for disease progression and spermatogenic impairment in VC.

Accumulating evidence indicates that dysregulation of multiple miRNAs was associated with male infertility 42. A recent study discovered that lycopene reduced hypoxia-induced damage to spermatocytes via the miR-23a/b-PROK2 axis 43. Ma et al. found that seminal exosomal miR-210-3p remarkably elevated in VC patients and miR-210-3p had a negative correlation with sperm count 44. Xu et al. discovered that miR-210-3p in seminal plasma triggered apoptosis in spermatogenic cells by stimulating caspase-3 activity in individuals with VC 45. However, reports on other small RNAs, such as piRNAs, in the development of VC are rare. In the present study, we discovered that piRNA-46403 was upregulated in testicular tissues of VC patients with asthenospermia and restrained spermatogonial proliferation by inducing Sertoli cell ferroptosis.

From a translational perspective, the identification of piRNA-46403 as a key regulator of Sertoli cell ferroptosis highlights its potential clinical value in the diagnosis and treatment of VC-associated male infertility. Given its significant upregulation in testicular tissues from VC patients, particularly those with impaired spermatogenesis, piRNA-46403 may serve as a molecular biomarker to stratify disease severity or predict spermatogenic dysfunction. From a therapeutic standpoint, targeted inhibition of piRNA-46403 may represent a novel approach to alleviate Sertoli cell ferroptosis and preserve spermatogenic function in VC patients. Furthermore, modulation of the downstream YBX1/m5C-modified USP18 axis could provide additional intervention points for preventing ferroptosis-driven testicular damage. Although further clinical validation and safety evaluation are required, our findings lay a foundation for the development of piRNA-based diagnostic tools and targeted therapies for VC-related male infertility.

Although our study identified piRNA-46403 as a key regulator of Sertoli cell ferroptosis in VC, the upstream mechanisms responsible for its aberrant expression remain unclear. VC is characterized by testicular hypoxia, oxidative stress, hyperthermia, and chronic inflammation. Previous studies have demonstrated that hypoxia is a prominent pathological feature of VC and contributes to testicular dysfunction through activation of HIF-1α-dependent signaling pathways 46, 47. Given that HIFs regulate the transcription of numerous coding and non-coding RNAs under stress conditions 48, 49, it is conceivable that hypoxia-associated transcriptional programs may directly or indirectly drive the upregulation of piRNA-46403 in Sertoli cells. Another possibility is that the genomic locus of piRNA-46403 is located within a stress-responsive regulatory region, rendering it susceptible to pathological activation. Future studies are warranted to investigate the genomic origin and transcriptional regulation of piRNA-46403 in the VC microenvironment, which may provide further insights into the pathogenesis of VC-associated infertility.

piRNAs are a class of small non-coding RNAs that predominantly associate with PIWI proteins to regulate germline development 29, 50. In the classical paradigm, germline piRNAs function as a genome defense system, forming PIWI-piRNA complexes that silence transposable elements and thereby safeguard genomic integrity across generations 51, 52. This PIWI-dependent pathway is evolutionarily conserved and represents a fundamental mechanism for protecting germline stability through transcriptional and post-transcriptional repression of mobile genetic elements. For example, the Piwi-piRNA complex initiated transposon silencing through transcription termination factors PNUTS and Senataxin 53. Cardiac myofibroblast-derived small extracellular vesicles-derived piRNA-62788 attenuated fibrotic responses by PIWIL2-mediated SRF silencing 54. However, emerging evidence has challenged this strictly germline-restricted and PIWI-centric view of piRNA biology. In somatic tissues or disease contexts, piRNAs may no longer primarily function in transposon silencing but instead participate in gene regulatory networks through PIWI-independent mechanisms 55. Some non-canonical piRNAs can act as direct protein interactors or molecular decoys, functionally resembling long non-coding RNAs by binding RNA-binding proteins or signaling mediators to modulate post-transcriptional regulation. For example, piR-bmo-796514 promotes the replication of the exogenous DNA virus baculovirus through targeting the E3 ubiquitin ligase RNF181 56. PiRNA CFAPIR repressed cardiac fibrosis by interacting with the muscleblind-like protein MBNL2 57. To investigate the regulatory mechanism of piRNA-46403 in VC-associated spermatogenic dysfunction, we identified its binding proteins via RNA pull-down coupled with mass spectrometry. However, no enrichment of classical PIWI protein was detected in our results. piRNA-46403 overexpression neither affected LINE1 transcription nor altered its methylation status. This finding suggests that piRNA-46403 may not function through the canonical PIWI-piRNA complex, but rather through a lncRNA-like manner. Specifically, we demonstrate that piRNA-46403 contributes to VC-related infertility through a mechanism independent of transposon regulation. We discovered that piRNA-46403 bound to YBX1 and disrupting its interaction with m5C-modified USP18 mRNA, reducing USP18 mRNA stability. Functionally, this axis contributes to the regulation of Sertoli cell ferroptosis. Collectively, our findings suggest that piRNA-46403 represents a non-canonical, PIWI-independent piRNA that functions as a protein decoy rather than a transposon silencer, thereby expanding the emerging role of somatic piRNAs in regulating stress responses and reproductive pathology.

Post-transcriptional RNA modifications are widely involved in male infertility. Aberrant expression of multiple m6A regulatory factors has been reported in human semen samples with oligospermia, asthenospermia, and azoospermia, contributing substantially to spermatogenic defects 58, 59. However, whether m5C modification, another key RNA modification, plays a role in VC-mediated infertility has not yet been fully elucidated. In this study, we found that piRNA-46403 could bind to YBX1, an m5C reader. It is worth noting that YBX1 can recognize and bind to RNA, and is involved in almost all RNA-related biological activities, including RNA splicing, stability maintenance, and translation 60, 61. Furthermore, although DNMT1 has been traditionally characterized as a DNA methyltransferase, emerging evidence suggests that DNMT1 is capable of catalyzing m5C modification on specific RNA substrates, thereby regulating RNA stability and post-transcriptional gene expression 62, 63. These findings provide mechanistic support for our observation that DNMT1 mediates m5C modification of USP18 mRNA and contributes to its YBX1-dependent stabilization.

There are several limitations in our study. First, the limited sample size of clinical specimens is as a major limitation of this study. Future validation in larger, multicenter cohorts is necessary to confirm our findings and assess the biomarker potential of piRNA-46403. Second, although we elucidated the YBX1/m5C modified-USP18 axis as a key downstream mechanism of piRNA-46403, the upstream factors driving piRNA-46403 dysregulation in the VC-mediated infertility microenvironment remain unclear. Third, this study primarily focused on Sertoli cells; however, VC involves complex interactions among multiple testicular cell types, and whether piRNA-46403 exerts additional effects in other cellular compartments warrants further investigation. Fourth, although functional studies in cellular and animal models support the pathogenic role of piRNA-46403, these models may not fully recapitulate the chronic and heterogeneous nature of human VC. Fifth, we primarily focused on the ferroptosis-regulatory function of the piRNA-46403/YBX1/USP18 axis. Reactome analyses also highlighted enrichment of Interferon signaling and Immune system, raising the possibility that piRNA-46403 may participate in broader immune and stress-response processes. Future studies are needed to determine whether these pathways contribute to VC-associated testicular dysfunction. Finally, while our data suggest translational potential, the feasibility, specificity, and safety of therapeutically targeting piRNA-46403 or the YBX1/m5C modified-USP18 pathway in humans require further rigorous preclinical and clinical evaluation.

In conclusion, our study disclosed that piRNA-46403 was upregulated in testicular tissues of VC patients with asthenospermia. Mechanistically, we provide novel evidence that piRNA-46403 promotes Sertoli cell ferroptosis by repressing USP18 mRNA stability in an m5C-dependent manner. These findings suggest that piRNA-46403 may serve as a potential therapeutic target for the treatment of VC-mediated infertility.

## Supplementary Material

Supplementary figures and tables.

## Data Availability

All data supporting the findings of this study are available within the main text and Supplementary Materials.
