# Supplementary material for: piRNA-46403 Promotes Sertoli Cell Ferroptosis via Disrupting m5C-dependent USP18 mRNA Stabilization in Varicocele-mediated Infertility
Source: Int J Biol Sci. 2026 Aug 12;22(13):7295–315. doi: 10.7150/ijbs.137298 (PMC13540690; doi:10.7150/ijbs.137298)
Supplement: Supplementary file 1 [file ijbsv22p7295s1.pdf]

Supplementary Figures

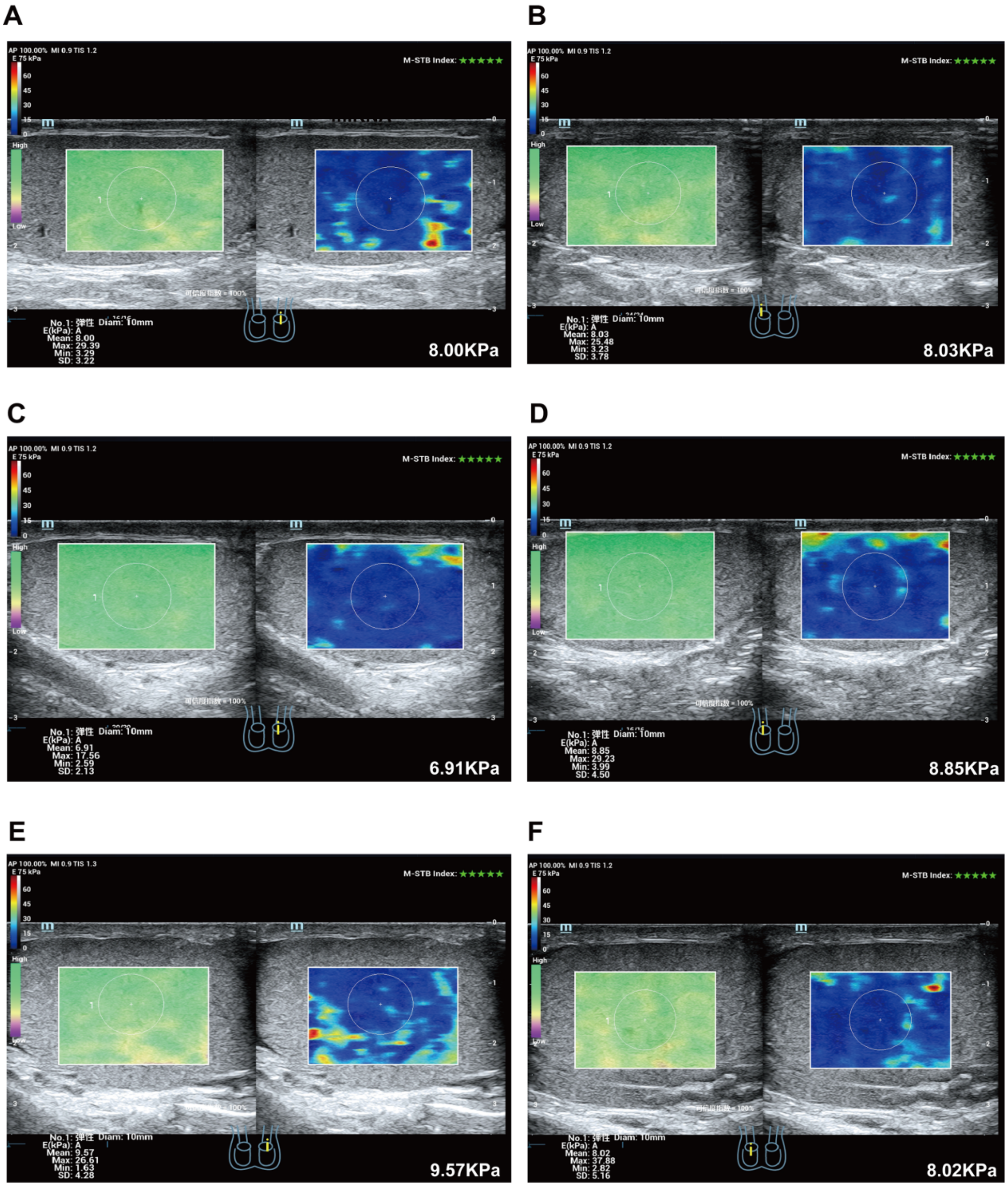

Figure S1 Correlation between varicocele (VC) severity and testicular elasticity. The association between VC severity and testicular elasticity in NC controls (A, B), left-sided VC with normal spermogram parameters (C, D), and left-sided VC with abnormal spermogram parameters (E, F).

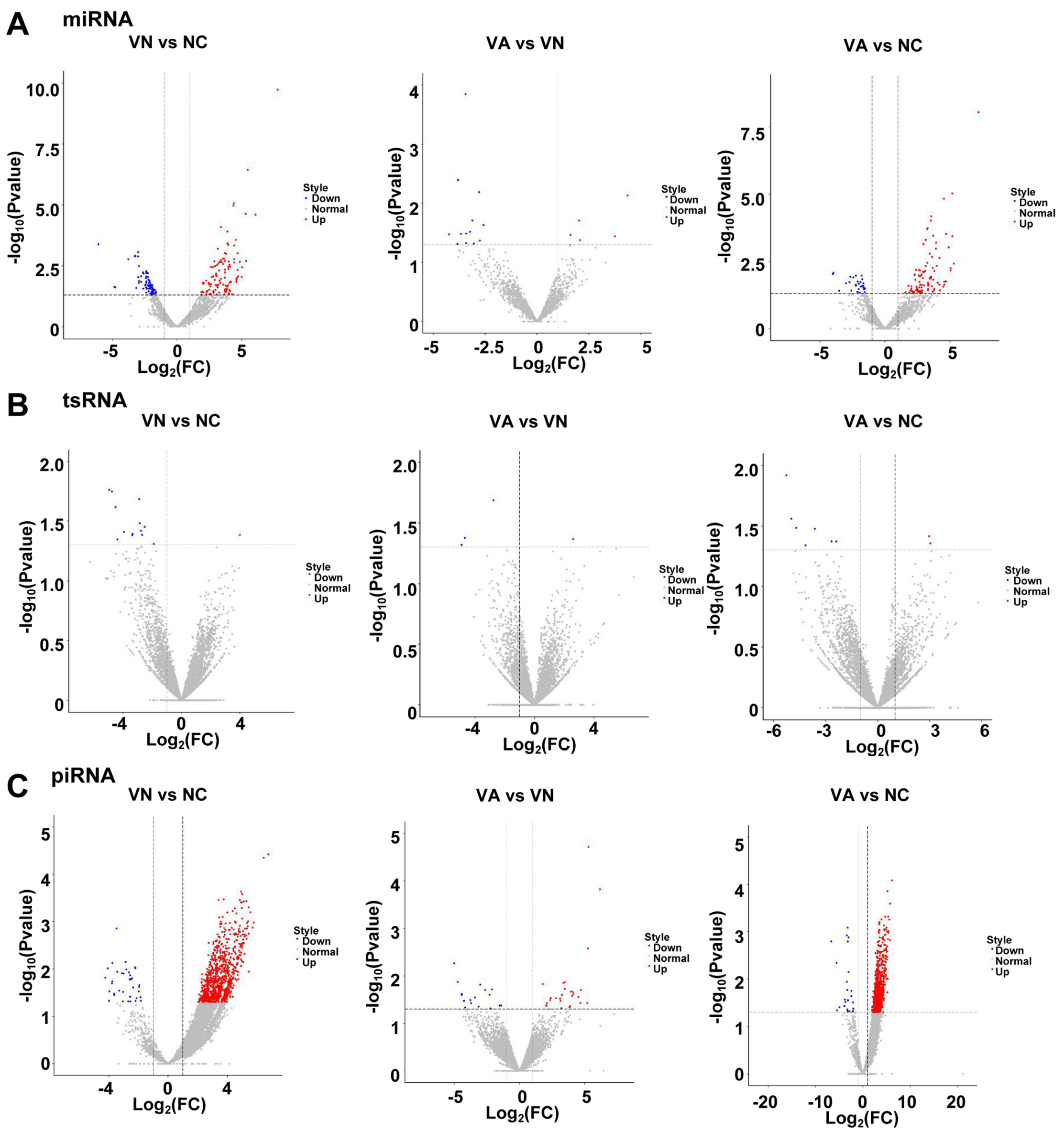

Figure S2 Identification of differentially expressed small RNAs in the testicular tissues of patients with varicocele (VC). Volcano plot of differentially expressed miRNAs (A), tsRNAs (B), and piRNAs (C). NC represents testicular tumor patients undergoing radical orchiectomy with WHO-normal semen parameters, from whom histologically normal adjacent testicular tissues were obtained. VN represents VC patients with WHO-normal semen parameters, and VA represents VC patients with asthenospermia, with testicular tissues collected accordingly.

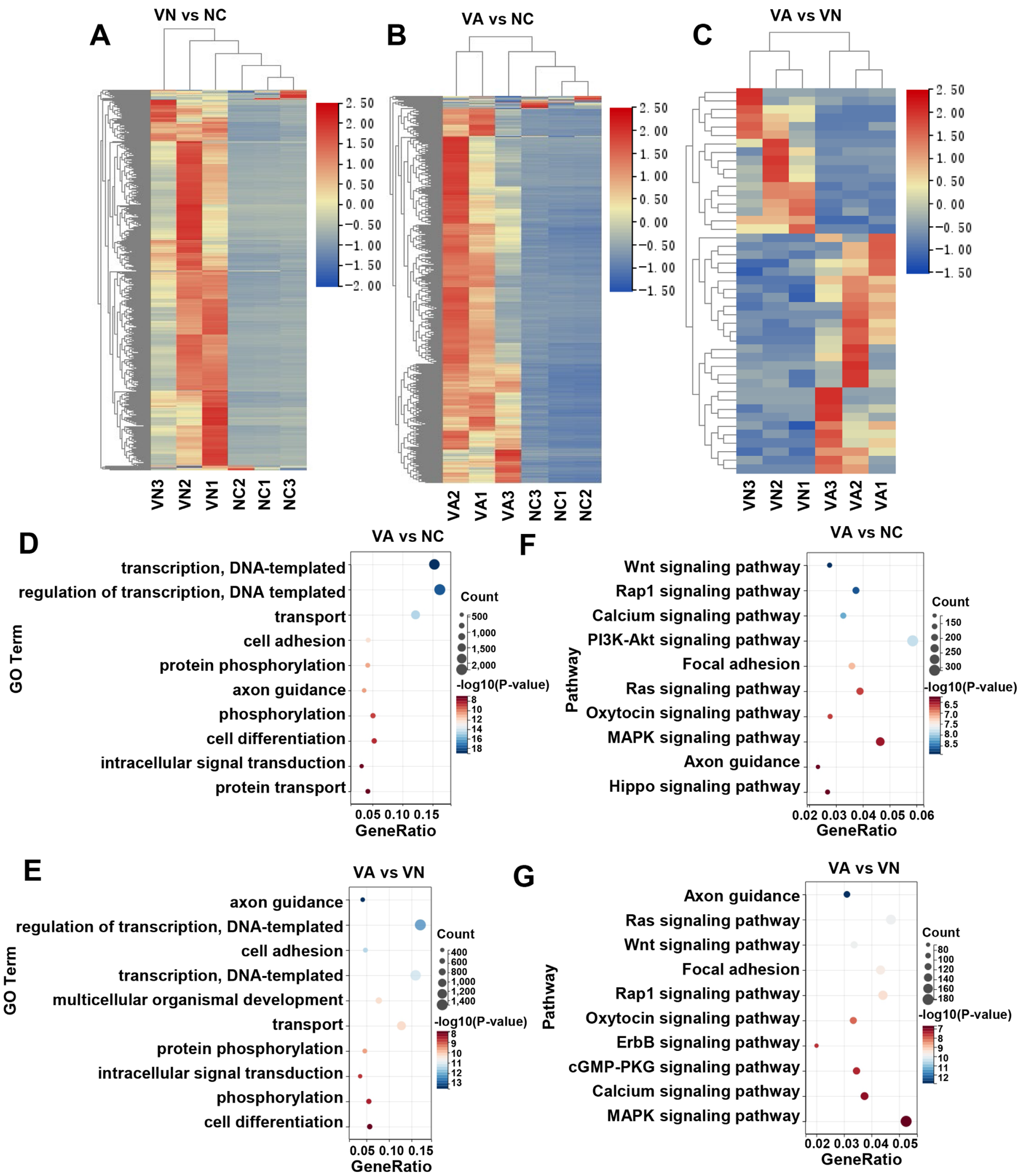

Figure S3 Functional analysis of differentially expressed piRNAs. (A-B) Heatmap of differentially expressed piRNAs in the VN vs NC group (A), VA vs NC group (B), and VA vs VN group (C). (D, E) GO analysis of differentially expressed piRNAs between the VA and NC (D) or VN (E) groups. (F, G) KEGG pathway enrichment analysis of differentially expressed piRNAs between the VA and NC (F) or VN (G) groups.

A

Sequence annotation of piRNA-46403

**Organism**	*Homo sapiens*
**Length**	31
**Sequence**	TCTTCTCGGAGCTGCTGTCCAAACTGTACTC
**piRNA loci**	chr15 : 101762373 - 101762404 (+)
**Genome Assembly**	GRCh38/hg38
**%GC content**	51.61
**Nucleotide Bias**	1T 10A
**Alias ID**	gi|108062927, DQ578291.1, piR-46403

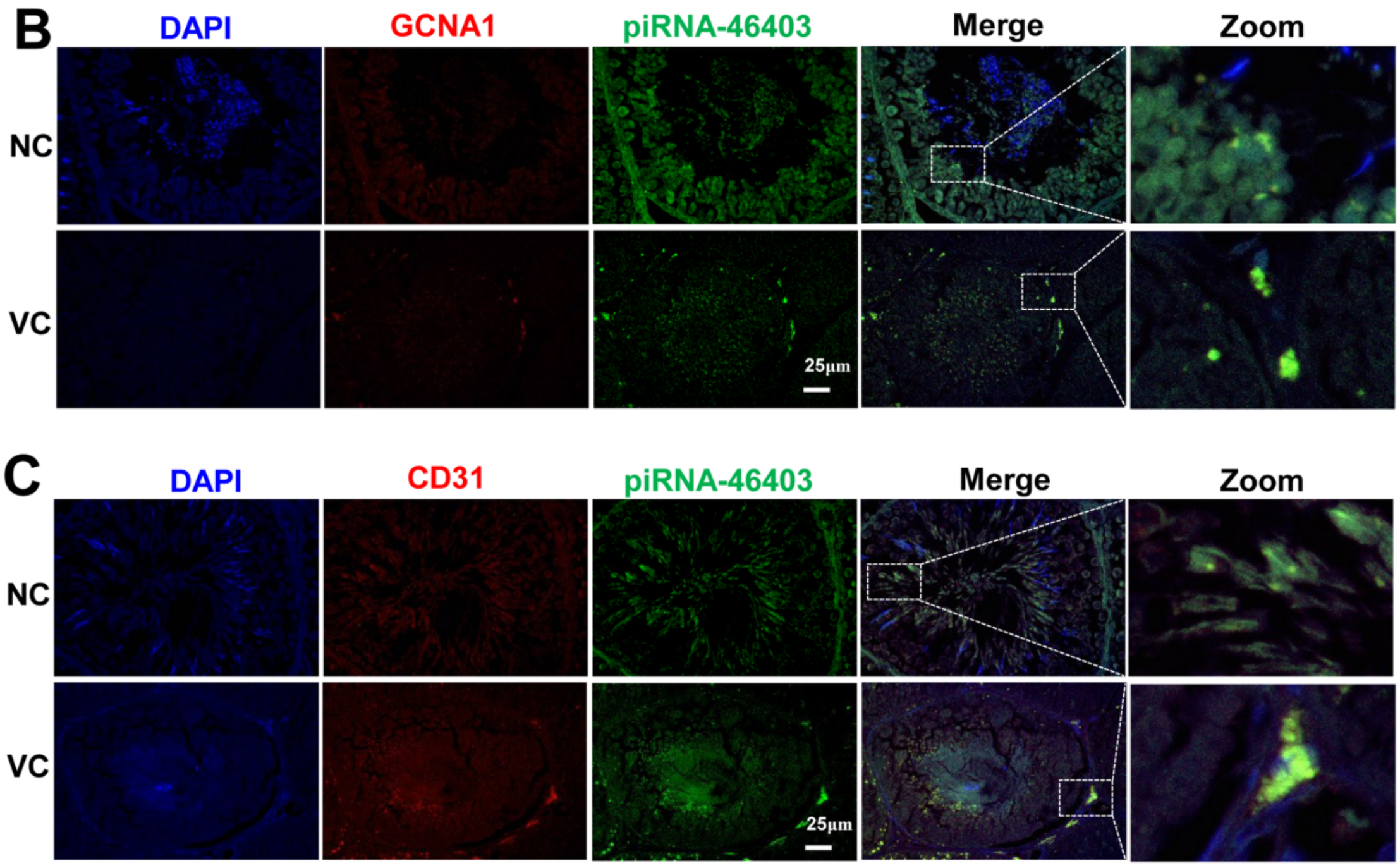

Figure S4 The expression of piRNA-46403 in spermatogonia (marker GCNA1) and endothelial cells (marker CD31) of testicular tissues. (A) Sequence characteristics of piRNA-46403. (B) Immunofluorescence and FISH were utilized to assess co-localization and expression of piRNA-46403 and GCNA1 in testicular tissues. (C) Immunofluorescence and FISH were used to detect co-localization and expression of piRNA-46403 and CD31 in testicular tissues. Scale bar=25μm.

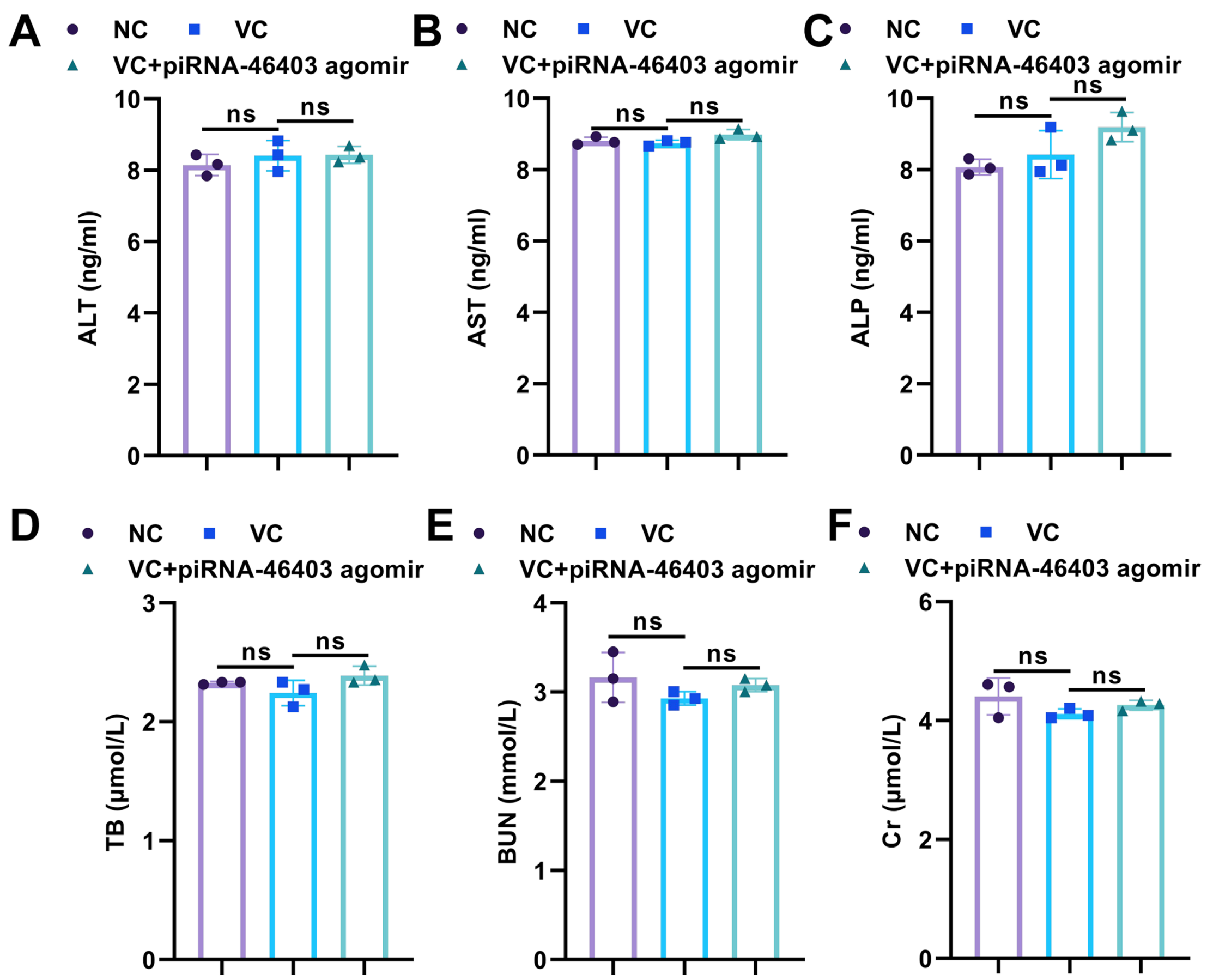

Figure S5 Effects of piRNA-46403 overexpression on liver and renal function in a varicocele (VC) rat model. (A-F) Serum levels of liver function markers (A) ALT, (B) AST, (C) ALP, (D) TB, and renal function markers (E) BUN, and (F) Cr, were measured. ns, not significant.

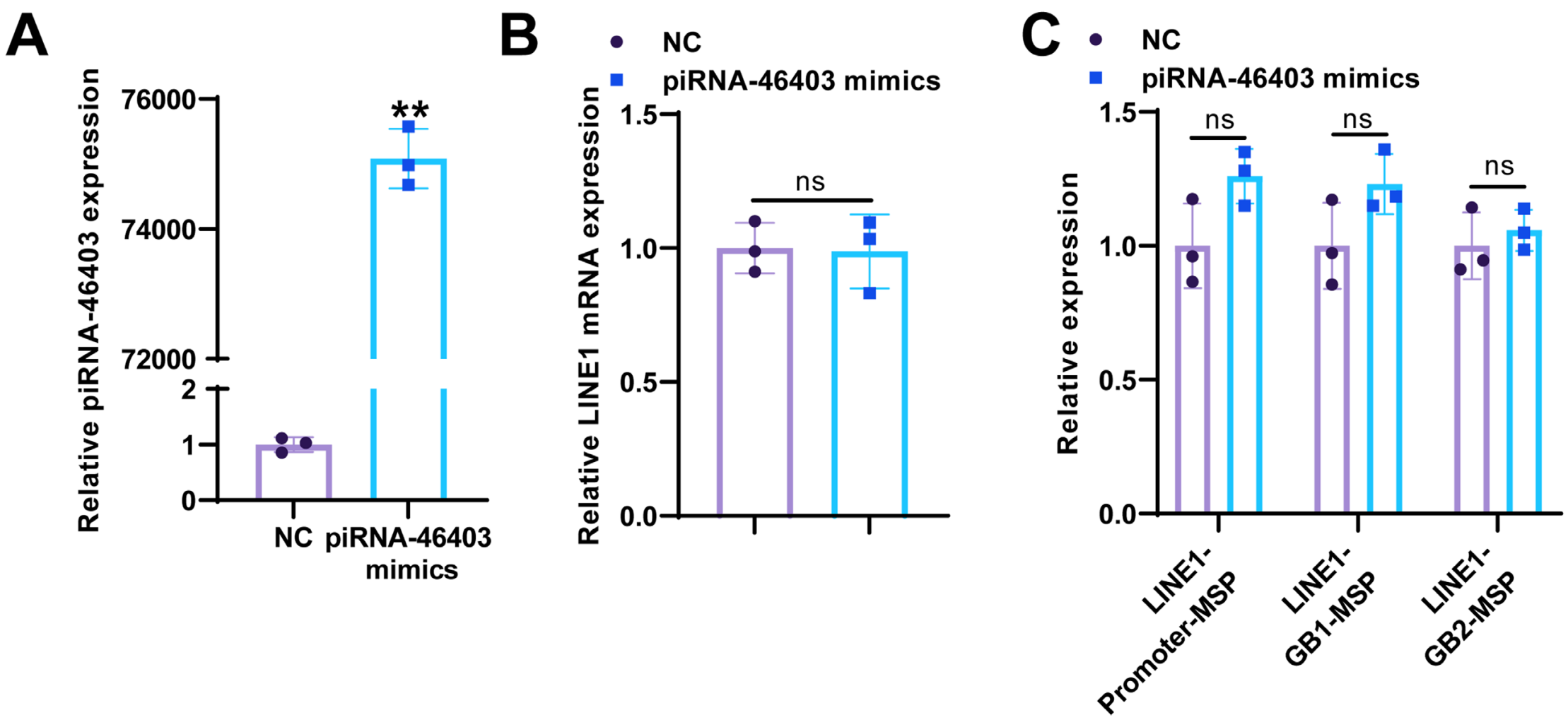

Figure S6 The LINE1 expression and methylation levels in piRNA-46403-overexpressed Sertoli cells. (A) qRT-PCR analysis of the overexpression efficiency of piRNA-46403. (B) The effect of piRNA-46403 overexpression on LINE1 expression in Sertoli cells was assessed by qRT-PCR. (C)

LINE1 methylation levels in Sertoli cells following piRNA-46403 overexpression were evaluated by qRT-PCR. ns, not significant, **P<0.01.

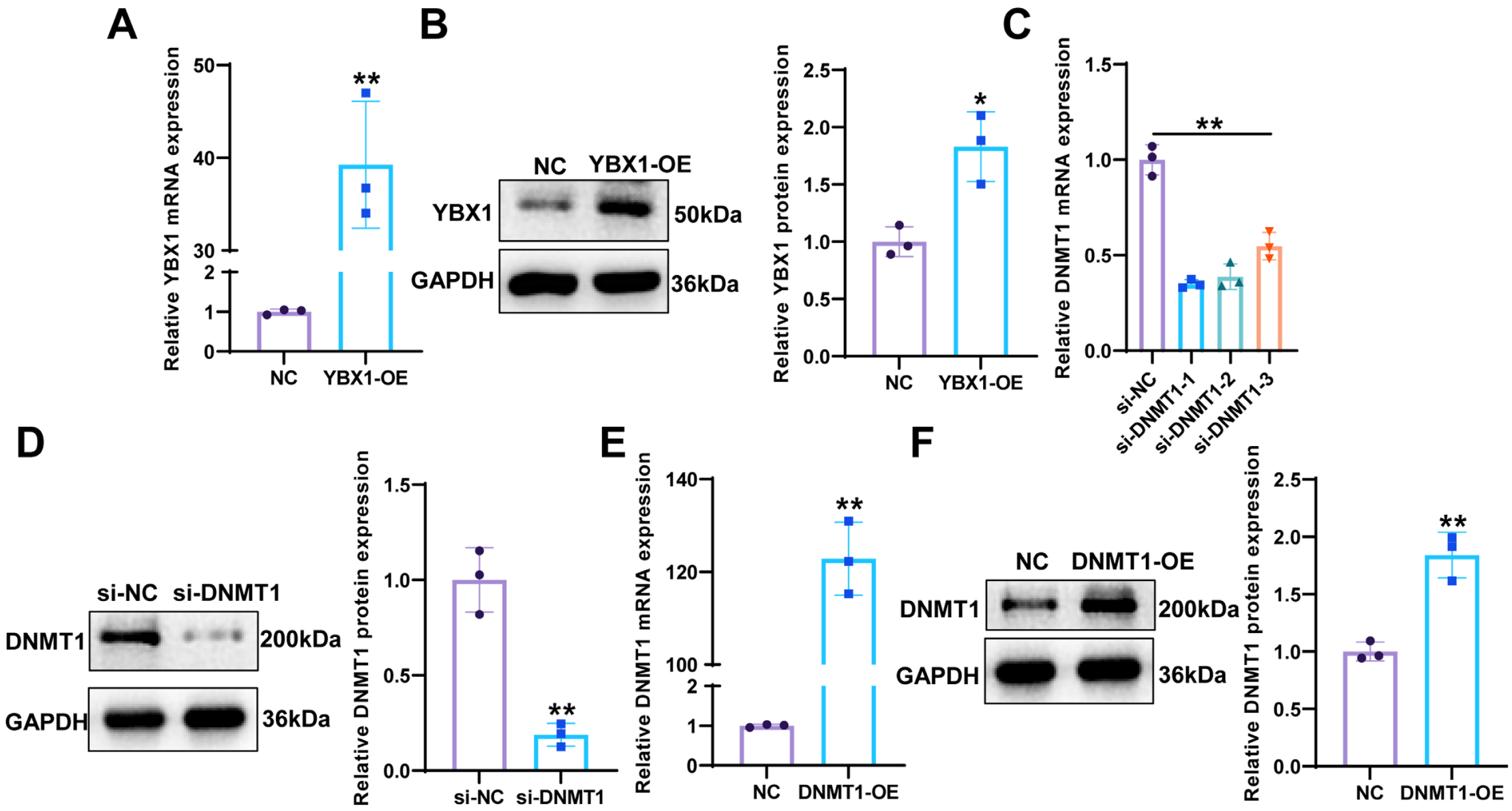

Figure S7 Verification of piRNA-46403 and YBX1 overexpression, as well as DNMT1 knockdown and overexpression, in Sertoli cells. (A, B) The overexpression efficiency of YBX1 was verified by qRT-PCR (A) and western blot (B). (C, D) qRT-PCR and western blot confirmed the knockdown efficiency of DNMT1. (E, F) qRT-PCR and western blot were used to validate the overexpression efficiency of DNMT1. *P<0.05; **P<0.01.

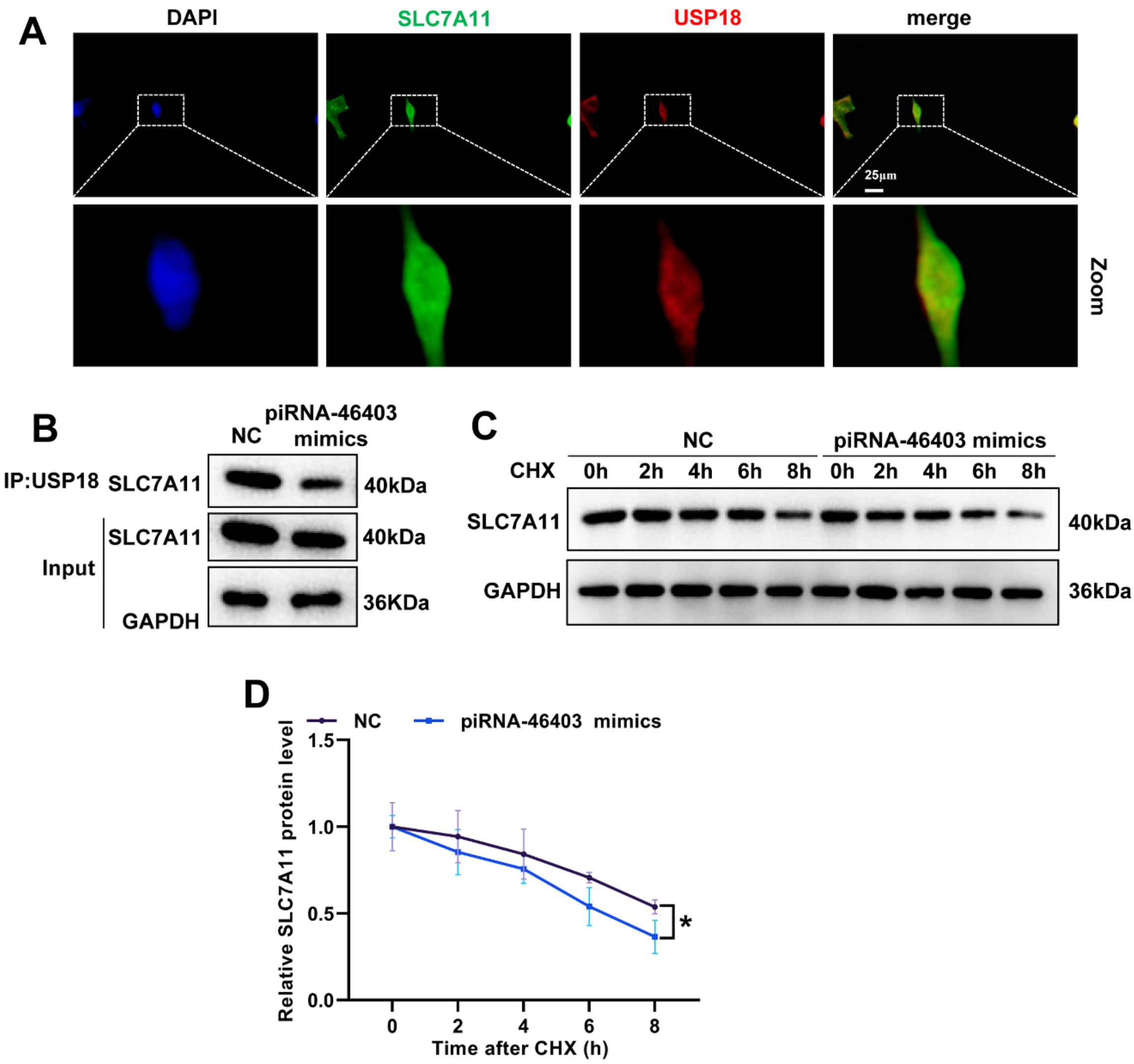

Figure S8 piRNA-46403 regulates SLC7A11 protein stability via USP18. (A) Immunofluorescence was performed to detect the subcellular localization of USP18 and SLC7A11. (B) Co-immunoprecipitation was used to examine the interaction between USP18 and SLC7A11. (C, D) Protein stability of SLC7A11 was assessed by CHX chase assay. *P<0.05.

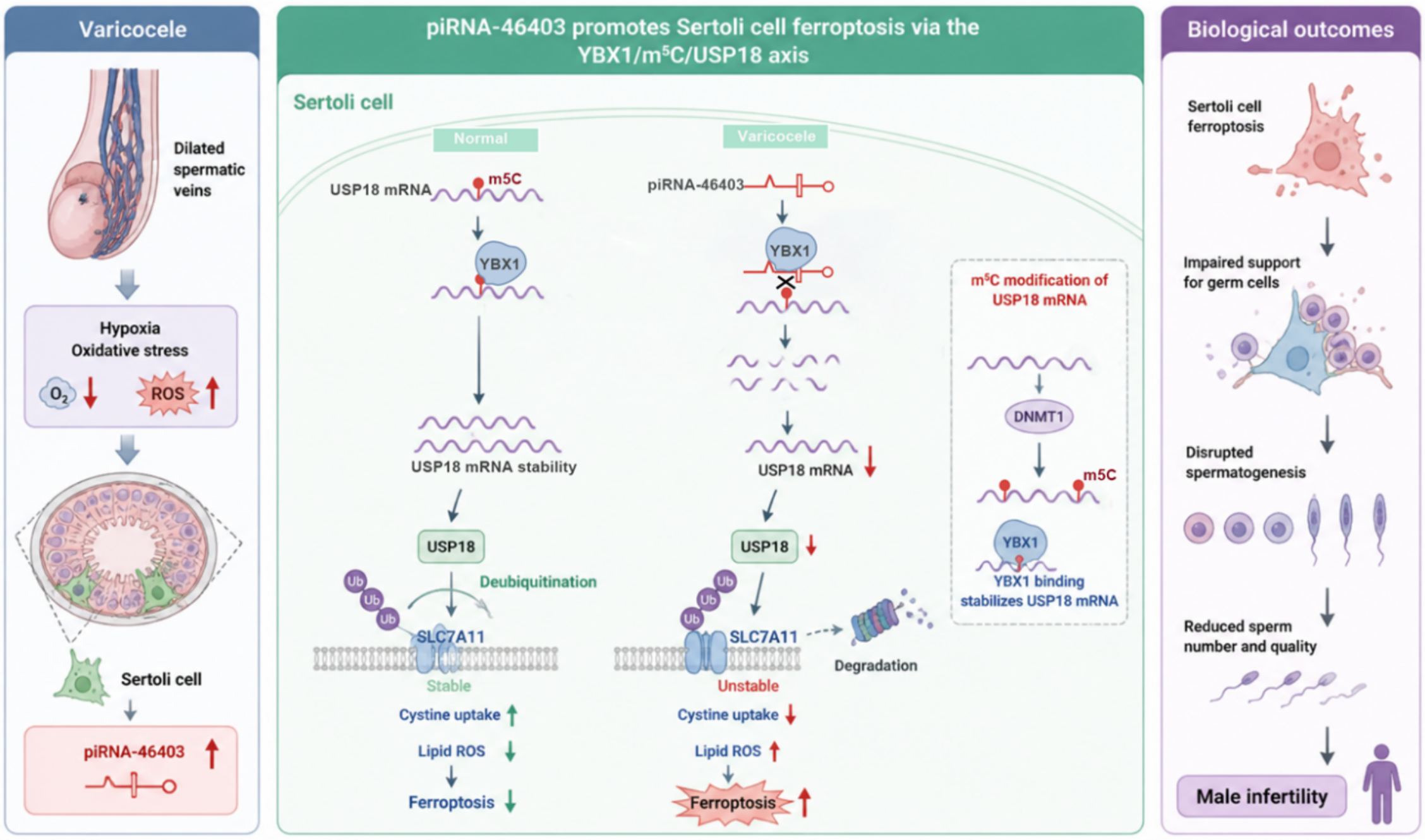

Figure S9 Schematic diagram of piRNA-46403/YBX1/USP18 axis regulating Sertoli cell ferroptosis in varicocele-mediated male infertility.

Table S1 Clinical characteristics of the participants

Characteristics	NC (n=5)	VN (n=7)	VA (n=10)	P value (NC vs VN)	P value (NC vs VA)	P value (VN vs VA)
Age (years)	23.60±2.97	22.43±4.28	25.10±3.90	0.8628	0.7600	0.3570
sperm concentration (M/ml)	87.86±50.50	41.66±13.43	63.51±54.75	0.2103	0.5915	0.5930
Total sperm motility (%)	53.00±5.20	56.86±6.59	36.20±13.40	0.7976	0.0192	0.0017
Forward motility of sperm (%)	43.60±6.35	42.86±5.67	22.20±8.15	0.9825	<0.0001	<0.0001
abnormal morphology (%)	89.40±7.37	89.86±1.86	95.90±1.52	0.9757	0.0121	0.0096

Note: NC represents testicular tumor patients undergoing radical inguinal orchiectomy, with preoperative semen parameters within WHO-normal reference ranges, VN represents VC patients with normal semen, VA represents VC patients with asthenospermia.

Table S2 Primer sequences

Primers	Sequence (5' to 3')
U6-F	CGATACAGAGAAGATTAGCATGGC
U6-R	AACGCTTCACGAATTTGCGT
piRNA-58317-F	ggacctgcctcttggt
piRNA-58317-RT	GTCGTATCCAGTGCGTGTCGTGGAGTCGGCAATTGCACTGGATACGACACATGAT
piRNA-36388-F	gatcaagatgcctgaggattc
piRNA-36388-RT	GTCGTATCCAGTGCGTGTCGTGGAGTCGGCAATTGCACTGGATACGACAGGCACT
piRNA-34615-F	gagatcaagatgcctgagg
piRNA-34615-RT	GTCGTATCCAGTGCGTGTCGTGGAGTCGGCAATTGCACTGGATACGACGGCACT
piRNA-42954-F	gcagtccatctgtgcaag
piRNA-42954-RT	GTCGTATCCAGTGCGTGTCGTGGAGTCGGCAATTGCACTGGATACGACGGTGTGG
piRNA-46403-F	gtcttctcggagctgct
piRNA-46403-RT	GTCGTATCCAGTGCGTGTCGTGGAGTCGGCAATTGCACTGGATACGACGAGTACA
General primer-R	AGTGCGTGTCGTGGAGTCG
GAPDH-F	ACAACTTTGGTATCGTGGAAGG
GAPDH-R	GCCATCACGCCACAGTTTC
USP18-F	AACGTGCCCTTGTTTGTCCAA
USP18-R	GAGTCCTTCACCCGGATCGTA
YBX1-F	CCCCAGGAAGTACCTTCGC
YBX1-R	AGCGTCTATAATGGTTACGGTCT
DNMT1-F	CCTAGCCCCAGGATTACAAGG
DNMT1-R	ACTCATCCGATTTGGCTCTTTC
L1-ORF1-F	GAATGATTTTGACGAGCTGAGAGAA
L1-ORF1-R	GTCCTCCCGTAGCTCAGAGTAATT
LINE1-Promoter-MSP-F	GTCGAATAGGAATAGTTTCGG
LINE1-Promoter-MSP-R	ACTCCCTAACCCCTTACGCT
LINE1-GB1-MSP-F	GTTTAGATTTAGGAAATATAGAGAAC
LINE1-GB1-MSP-R	CTAACTTATAAAATTTCTACCGAA
LINE1-GB2-MSP-F	GTTGGATGGAGAATGATTTTGAC
LINE1-GB2-	TTAATCACATCGACTCCTAAAAC

MSP-R	
Actin-MSP-F	TGGTGATGGAGGAGGTTTAGTAAG
Actin-MSP-R	AACCAATAAAACCTACTCCTCCCTTAA

Table S3 Pull-down combined with mass spectrometry to identify proteins bound to piRNA-46403

Accession	Coverage [%]	Peptides	PSMs	Unique Peptides	AAs	MW [kDa]	calc. pI	Abundances (Grouped): F1
H6VRF8	52	33	56	28	644	66	8.12	759192329.9
P35527	51	28	38	27	623	62	5.24	271530654.2
A0A1B0GVI3	51	26	39	23	624	63.3	5.26	232855905.2
A0A024R0Y2	16	31	34	31	2268	257.1	6.61	27643788.25
P35908	38	22	30	14	639	65.4	8	129316770.4
Q15149	8	34	34	34	4684	531.5	5.96	13213479.12
A0A804HL40	21	26	27	26	1393	157.7	6.18	27928447.44
Q92945	35	23	25	19	711	73.1	7.3	78753119.78
A6NLN1	31	10	18	8	527	56.5	9.38	124854796.6
Q969I0	59	21	28	15	365	41.1	5	66954764.25
A0A7I2V4F0	31	18	20	18	734	81.7	7.33	23865309.06
P23246	25	14	19	13	707	76.1	9.44	88229323.83
P19338	28	18	20	18	710	76.6	4.7	66896886
P13647	29	19	22	10	590	62.3	7.74	22766705.91
B2R853	29	17	19	5	564	60	7.72	6123293.625
P02768	14	10	21	10	609	69.3	6.28	3319934306
P02533	36	14	16	5	472	51.5	5.16	21424679.56
Q96AE4	26	16	17	11	64	67.5	7.61	19547498.47

					4			
Q2TSD0	36	9	11	9	335	36	8.46	19193172.47
A0A7I2V535	21	11	17	10	501	57.6	9.33	15979546.06
A0A7I2YQN2	22	12	14	12	561	62.5	7.56	21214001.88
P78386	28	16	18	8	507	55.8	6.55	69008349.78
Q86YZ3	9	9	10	9	2850	282.2	10.04	9559612.938
P08727	28	13	15	6	400	44.1	5.14	15771512.22
Q6FI03	23	8	11	7	466	52.1	5.59	16913512.38
O95678	13	9	11	2	551	59.5	7.74	13207487.13
A0A087X0X3	19	13	13	13	730	77.5	8.78	10805741.88
Q96I24	20	11	12	8	572	61.6	8.38	7448383.031
E9PKE3	21	10	10	8	627	68.8	5.52	7530984.656
A0A140TA69	19	10	11	4	436	49.4	5.06	50078611.75
Q53HQ7	21	8	10	8	462	50.2	8.94	24085858.75
B4DZC3	11	9	9	9	896	102.4	7.64	4834669.141
A0A0G2JLI4	17	9	9	6	509	57.1	8.24	6063420.156
Q14525	15	8	10	3	404	46.2	4.84	13125512.41
Q14533	15	8	10	2	505	54.9	5.47	3907576.125
P05783	19	9	9	8	430	48	5.45	18184241.25
A0A087WZ13	19	9	10	9	739	77.8	8.84	3733954.656
B4DUQ1	23	8	8	8	439	48.5	5.92	7875947.281
Q53G99	24	8	8	8	375	41.7	5.59	7452057.281
A0A024R	12	4	6	3	44	50.8	5.38	4886935.25

DB2					9			
P08779	17	8	9	1	473	51.2	5.05	527927.3125
Q04695	15	8	9	2	432	48.1	5.02	496210.0938
Q92764	11	6	8	2	455	50.3	4.91	2851267.844
P04075	23	6	6	6	364	39.4	8.09	4927416.906
Q9Y6M1	13	7	7	7	599	66.1	8.46	3585028.406
A0A7G6J4N4	12	8	8	8	802	87.5	5.27	4247734.719
A0A024RDF4	22	7	8	5	306	32.8	8.16	13384592.19
A0A5H1ZRQ2	8	6	6	3	731	80.4	8.37	1663879.406
Q7Z794	5	3	6	1	578	61.9	5.99	347366.1563
Q14444	10	6	7	6	709	78.3	5.25	9552723.094
Q9Y3I0	14	5	5	5	505	55.2	7.23	2367852.266
B2R6U8	25	5	5	5	227	26.2	8.82	4009029.563
Q14532	11	5	6	2	448	50.3	4.84	975876.2188
P84103	20	4	5	3	164	19.3	11.65	8565725.313
Q00341	6	6	6	6	1268	141.4	6.87	1996568.063
P11498	6	6	6	6	1178	129.6	6.84	1869713.688
Q6FHZ0	13	5	5	5	338	35.5	8.68	2120885.969
A0A1X7SBS1	8	5	5	5	744	81.7	5.83	4873667.188
Q9UHD8	11	6	6	6	586	65.4	8.97	2656234.406
O95758	9	5	5	3	552	59.7	9.04	1776988.688
P05141	14	4	4	4	298	32.8	9.69	1593723.688
A8MXP9	6	5	5	5	89	99.9	6.04	522080.4063

					5			
Q701L7	7	4	4	1	513	56.6	6.74	1110232.125
Q9GZT3	39	4	4	4	109	12.3	10.24	2358221.188
J3QTR3	25	3	13	3	106	12.2	10.14	92919872.25
Q16629	11	2	3	1	238	27.4	11.82	533313.0625
Q96IR1	19	5	5	5	243	27.2	9.94	1846677.781
D6W5Y5	11	3	3	3	297	31.9	9.61	1455926.75
A0A7I2V649	6	4	4	4	590	65.1	9.36	1429164.406
F8VZX2	14	4	4	4	321	33.8	8.24	2550056.781
A0A024QZ91	5	4	4	4	975	103.5	8.12	1268691.344
A0A7P0TAI0	9	4	4	2	616	68.1	5.14	677325.4688
P81605	26	3	3	3	110	11.3	6.54	16365822.19
A1A508	9	2	3	1	247	26.5	7.21	989542536.9
F8W1S1	4	3	3	1	543	59.4	7.44	904594.625
Q99729	8	3	3	1	332	36.2	8.21	
A8K6U7	6	4	5	4	856	95.6	6.92	706163.875
P15924	1	4	4	4	2871	331.6	6.81	1292559.594
D3DQ69	6	2	2	2	450	49.1	9.33	349826.625
Q4VB24	13	3	3	3	219	21.9	11.03	2596147.438
Q02413	3	2	2	2	1049	113.7	5.03	916593.75
Q8N5L9	13	4	4	4	293	31.3	10.24	1262010.719
Q5D862	1	2	2	2	2391	247.9	8.31	703872.125
E7EQ64	11	2	4	1	26	28.1	7.25	366418.3438

					1			
X6RDA4	18	3	3	3	248	27.3	5.45	1495273.844
Q96PK6	7	3	3	3	669	69.4	9.67	1395477.813
P08670	6	3	3	2	466	53.6	5.12	837176.2188
Q6NUR7	4	3	3	3	586	69.2	6.27	2152879.563
A0A0B4J259	15	2	2	2	137	15.3	8.69	2489623.625
Q01085	11	3	3	3	375	41.6	7.74	494706.75
P05109	24	2	3	2	93	10.8	7.03	2475366.313
P62805	31	3	3	3	103	11.4	11.36	1911921.969
P31151	23	2	3	2	101	11.5	6.77	1419167.5
Q9Y224	11	3	3	3	244	28.1	6.65	2851891.625
V9HWC6	14	3	3	3	208	22.7	9.32	1603954
P0DPH7	7	3	4	3	450	49.9	5.1	997579.75
P23396	9	2	2	2	243	26.7	9.66	485371.4219
Q8N684	4	2	2	2	471	52	8	816108.6875
A0A125QYY8	12	1	1	1	112	12.2	8.5	513955.2813
P62753	6	2	2	2	249	28.7	10.84	953792.875
E9PKN0	3	2	2	2	784	88.9	9.85	500066.3281
F8WJN3	5	2	2	2	478	52.2	6.43	2710251.125
Q16778	19	2	2	2	126	13.9	10.32	937561.5
B5BU25	4	2	2	2	471	53.1	9.09	450760.0625
P60866	19	2	2	2	119	13.4	9.94	1084546.5
P50991	5	3	3	3	539	57.9	7.83	439562.8281

Q53FN0	9	2	2	2	221	25.5	11.85	1844762.375
I6L9E8	6	2	2	2	518	55.1	8.88	989278.0313
E7EX73	2	2	2	2	1436	158.5	5.21	253488.7188
P08729	4	2	2	1	469	51.4	5.48	629484.1875
Q8TBR3	3	2	2	2	526	53.4	9.38	2413567.063
E9PB61	4	1	1	1	264	27.5	11.05	682230.5625
Q6PKI6	10	2	2	1	266	29.4	10.23	886473.4375
B4DYY5	2	2	2	2	1078	113.5	6.68	1121159.469
Q8N1N4	3	2	2	1	520	56.8	6.02	518363.4063
P16989	7	2	2	1	372	40.1	9.77	762863.2813
Q13151	7	1	1	1	305	30.8	9.29	1615472.25
Q96EP5	4	1	1	1	407	43.4	8.56	
C9JFR7	19	2	2	2	101	11.3	9.66	750118.7813
H0Y8K0	5	2	2	2	458	50	9.38	183438.5469
Q0QEL2	5	1	1	1	224	24.9	6.86	251276.625
P39748	5	2	2	2	380	42.6	8.62	791054.7813
B4DM21	4	2	2	2	490	55.1	7.72	1529679.094
Q75MU2	8	1	1	1	156	17.1	5.31	463932
A0A024RCT9	5	1	1	1	328	34.3	10.37	472889.1875
A0A3S8NFS6	2	1	1	1	414	47.1	6.04	379294.0938
M0QYW4	5	1	1	1	141	15.5	8.31	183389664
P26373	5	1	1	1	211	24.2	11.65	246923.5781

B0QYK0	1	1	1	1	618	64.9	9.38	2002922.875
P46783	5	1	1	1	165	18.9	10.15	
A0A024RA87	2	1	1	1	384	44.8	8.22	437596.1875
A2A3S3	3	1	1	1	361	39.3	8.43	516826.5313
Q08188	1	1	1	1	693	76.6	5.86	438664.5313
B2R5U1	1	1	1	1	885	99.6	6.96	244564.2969
Q16777	15	1	1	1	129	14	10.9	
Q9BTI9	4	1	1	1	228	25	4.86	592357.5
M0QX76	20	1	1	1	50	5.6	9.63	
Q92879	2	1	1	1	486	52	8.46	520293.625
C9J0E4	19	1	1	1	63	7.1	7.24	801828.875
Q8TA90	2	1	1	1	517	57.5	6.93	126749.2813
P26196	2	1	1	1	483	54.4	8.66	
E9PLX7	10	1	1	1	113	12.5	11.44	283013.4375
H0YNP5	6	1	1	1	175	19.7	5.21	496822.3125
B2RDE0	2	1	1	1	703	77.4	7.06	248896.7344
K7EMH1	15	1	1	1	89	10.4	9.66	356944.4688
A0A6Q8PF80	3	1	1	1	342	38.6	6.49	186693.4844
Q9UG93	3	1	1	1	369	40.6	5.86	353971.25
F5GXS0	1	1	1	1	1698	187.6	7.33	529410.6875
H7C2W9	8	1	1	1	108	12.8	11	601847.5
E9PFZ2	1	1	1	1	946	108.8	5.77	270034.8438
C9JH19	2	1	1	1	451	48.6	7.84	296249.5
Q6ZQN2	2	1	2	1	17	180.6	6.15	531994.6875

					14			
Q5T750	3	1	1	1	250	26.2	7.97	1145045.125
A0A2R8Y811	9	1	1	1	150	16.1	10.05	418193.9375
H0YHA7	8	1	1	1	167	19	11.78	172231.0156
Q13061	1	1	1	1	729	81.5	9.42	796374.3125
K7ENJ4	4	1	1	1	199	21.3	10.45	391937.6563
B5MCT8	5	1	1	1	139	16.6	11.06	494366.75
A8K4W0	3	1	1	1	264	29.9	9.73	
A0A8V8TMM5	3	1	1	1	344	37.4	6.39	591161.3125
A0A0J9YYL3	2	1	1	1	505	54.6	5.34	292391.6563
A0A024R7L5	1	1	1	1	1118	123	6.68	124068.5469
B3KP18	1	1	1	1	792	90.5	5.45	557374.8125
E5RI99	11	1	1	1	114	12.6	9.55	195426.8906
Q9UKV8	1	1	1	1	859	97.1	9.19	142599.5
P05089	3	1	1	1	322	34.7	7.21	
K7EJV9	8	1	1	1	170	19.4	10.48	431069
H0YEX5	3	1	1	1	315	35	4.81	195986.0156
B2R9K8	2	1	1	1	531	57.9	6.8	95819.09375
H7C2E8	8	1	1	1	102	11.6	7.88	196167.0469
Q8TB74	2	1	1	1	298	34.3	7.3	853710.6875
P27694	2	1	1	1	616	68.1	7.21	359885.25
H0Y7V4	0	1	1	1	4188	478.6	6.11	228409.9531
Q9Y663	3	1	1	1	40	44.9	9.5	3228489.75

					6			
Q68D27	3	1	1	1	575	63.9	6.87	214181.7188
Q96P63	2	1	1	1	405	46.2	5.53	
B4DF70	6	1	1	1	183	20.1	8.78	227575.7656
F4MH93	2	1	1	1	482	54.9	6.99	2479155.5
C9JD32	9	1	1	1	91	9.7	11.49	949472
A0A494C0Y9	3	1	1	1	280	29.5	9.74	153896.1094
L0R6G7	5	1	1	1	139	16.7	12.86	644164.3125
P25311	4	1	1	1	298	34.2	6.05	
P06702	7	1	1	1	114	13.2	6.13	323191.7188
M0R1H5	10	1	1	1	79	9.5	10.65	

Table S4 The binding ability of proteins

Protein	RNA_ID	Top_RNA_fragment	Interaction_Propensity	Z_score
DNMT1	NM.017414.4	1377-1452	49.93	1.62
DNMT3B	NM.017414.4	1377-1452	46.11	1.4
NSUN2	NM.017414.4	1377-1452	40.52	1.08
TRDMT1	NM.017414.4	1562-1637	26.38	0.26
NSUN7	NM.017414.4	1377-1452	25.84	0.23
NSUN3	NM.017414.4	1562-1637	17	-0.28
NSUN6	NM.017414.4	1377-1452	16.19	-0.32
NSUN4	NM.017414.4	1562-1637	14.62	-0.41
NSUN5	NM.017414.4	1562-1637	10.38	-0.66
